# Evaluation status of current and emerging minimally invasive robotic surgical platforms

**DOI:** 10.1007/s00464-023-10554-4

**Published:** 2023-12-20

**Authors:** M. Boal, C. Giovene Di Girasole, F. Tesfai, T. E. M. Morrison, S. Higgs, J. Ahmad, A. Arezzo, N. Francis

**Affiliations:** 1 The Griffin Institute, Northwick Park and St Marks Hospital, London, UK; 2 Wellcome/EPSRC Centre for Intervention and Surgical Sciences, University College London, London, UK; 3 Association of Laparoscopic Surgeons of Great Britain and Ireland (ALSGBI) Academy, London, UK; 4 University College London, London, UK; 5 Gloucestershire Hospitals NHS Foundation Trust, Gloucester, UK; 6 University Hospitals Coventry and Warwickshire, Coventry, UK; 7 Department of Surgical Sciences, University of Turin, Turin, Italy; 8 Yeovil District Hospital, Somerset NHS Foundation Trust, Yeovil, UK

**Keywords:** Robotics, Evaluation, Validation, Device, Innovation

## Abstract

**Background:**

The rapid adoption of robotics within minimally invasive surgical specialties has also seen an explosion of new technology including multi- and single port, natural orifice transluminal endoscopic surgery (NOTES), endoluminal and “on-demand” platforms. This review aims to evaluate the validation status of current and emerging MIS robotic platforms, using the IDEAL Framework.

**Methods:**

A scoping review exploring robotic minimally invasive surgical devices, technology and systems in use or being developed was performed, including general surgery, gynaecology, urology and cardiothoracics. Systems operating purely outside the abdomen or thorax and endoluminal or natural orifice platforms were excluded. PubMed, Google Scholar, journal reports and information from the public domain were collected. Each company was approached via email for a virtual interview to discover more about the systems and to quality check data. The IDEAL Framework is an internationally accepted tool to evaluate novel surgical technology, consisting of four stages: idea, development/exploration, assessment, and surveillance. An IDEAL stage, synonymous with validation status in this review, was assigned by reviewing the published literature.

**Results:**

21 companies with 23 different robotic platforms were identified for data collection, 13 with national and/or international regulatory approval. Of the 17 multiport systems, 1 is fully evaluated at stage 4, 2 are stage 3, 6 stage 2b, 2 at stage 2a, 2 stage 1, and 4 at the pre-IDEAL stage 0. Of the 6 single-port systems none have been fully evaluated with 1 at stage 3, 3 at stage 1 and 2 at stage 0.

**Conclusions:**

The majority of existing robotic platforms are currently at the preclinical to developmental and exploratory stage of evaluation. Using the IDEAL framework will ensure that emerging robotic platforms are fully evaluated with long-term data, to inform the surgical workforce and ensure patient safety.

**Graphical abstract:**

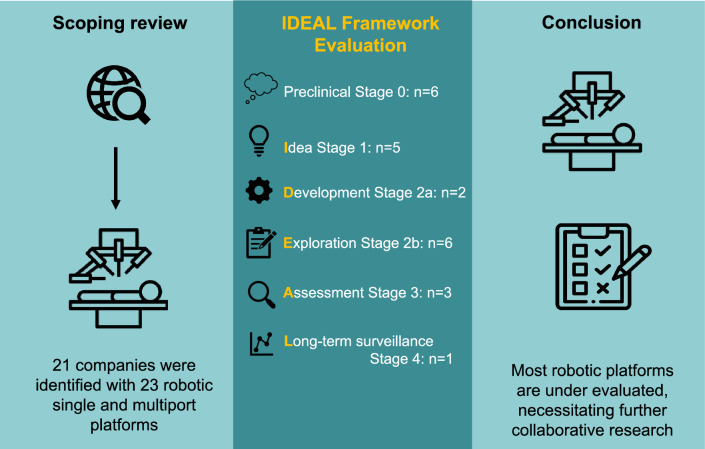

Excitement about robotic surgery continues to grow with the obvious benefits in surgeon ergonomics, high-definition 3D vision, dexterity, truly objective metrics for assessment, the application of artificial intelligence and augmented reality. Minimally invasive surgery (MIS) has evidence of non-inferiority in mortality outcomes compared to open surgery, but with superior outcomes in terms of patient morbidity and length of stay [1]. For robotic surgery, evidence is mounting as institutional learning curves are realised and long-term data becomes available, with improved patient outcomes when compared to laparoscopy in terms of morbidity [2–6] including lower blood loss, conversion-to-open, pain and shorter hospital stay. However, the evidence is mixed, for example in a meta-analysis comparing different approaches of total mesorectal excision in rectal cancer [7], and often equivalent outcomes in “smaller” operations [8, 9].

Roughly 82% of robotic surgery performed is within urology, general surgery, and gynaecology [10], but it is still only used in a minority of operations worldwide, due to availability and cost. To address this, many robotic platforms are in development or have recently reached the market, providing competition but also different approaches to broaden the capacity and capabilities of surgeons. As such there has been a rapid increase in robotic operations, one study of 73 hospitals stated an increase of 1.8% to 15.1% of all general surgery procedures were performed robotically between 2012 to 2018 [11] and the robotic surgery market globally was valued at $5.32 billion in 2019, estimated to grow to $19 billion in 2027 [12].

Along with this expansion there have been calls for reporting on safe implementation of novel platforms and standardisation of training and accreditation within robotics, due to concerns over errors and patient safety [13, 14]. Now the surgical community is faced with the additional challenge of evaluating multiple robotic platforms.

To our knowledge, there is no comprehensive, up-to-date review of existing platforms which can help guide the end-user, the surgeon, to decide which robot would be ideal for their purpose and the evidence to support it.

This scoping review aims to provide an update of current and emerging robotic platforms within minimally invasive surgical specialties, including a stage of evaluation using The Idea, Development, Explore, Assessment and Long-term study (IDEAL) Framework [15].

## Methods

A scoping review was performed, screening articles from PubMed, Google Scholar, journal reports, company websites and review articles. The search focused on minimally invasive robotic surgical platforms used within general surgery, gynaecology, urology, head and neck, cardiothoracics, given the application is predominantly in these specialties, and robots are broadly comparable in terms of function. Systems operating purely outside the abdomen or thorax and endoluminal or natural orifice platforms were excluded as these are potentially not comparable.

**Fig. 1:**
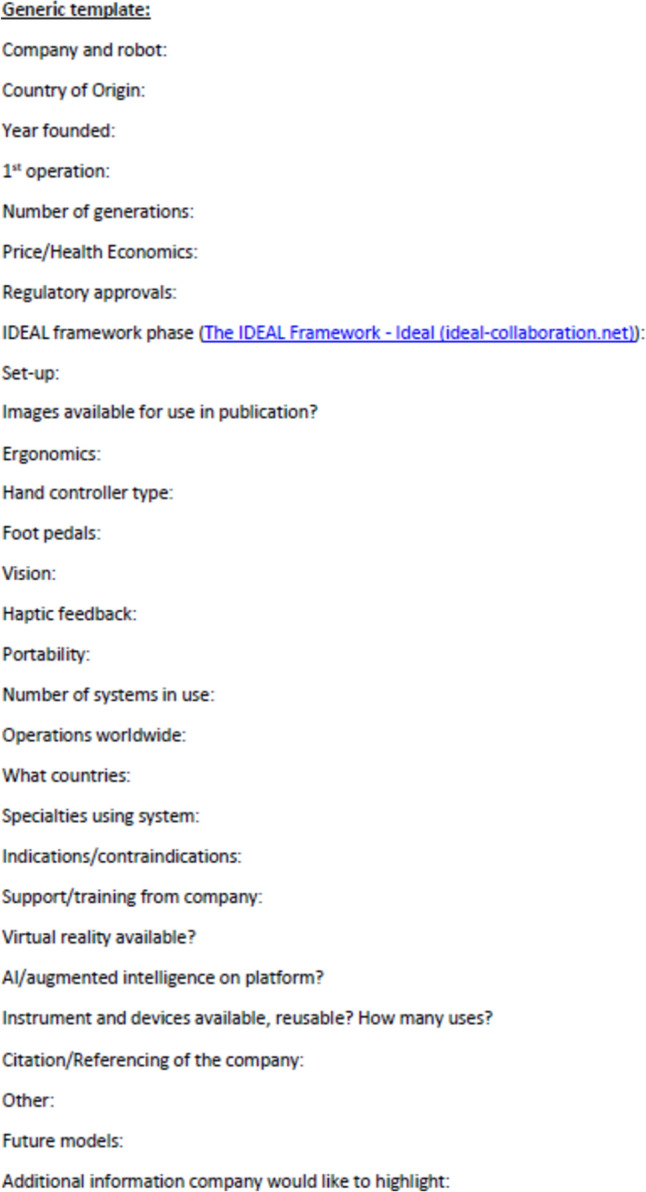
Virtual Interview/Data recording template

**Fig. 2:**
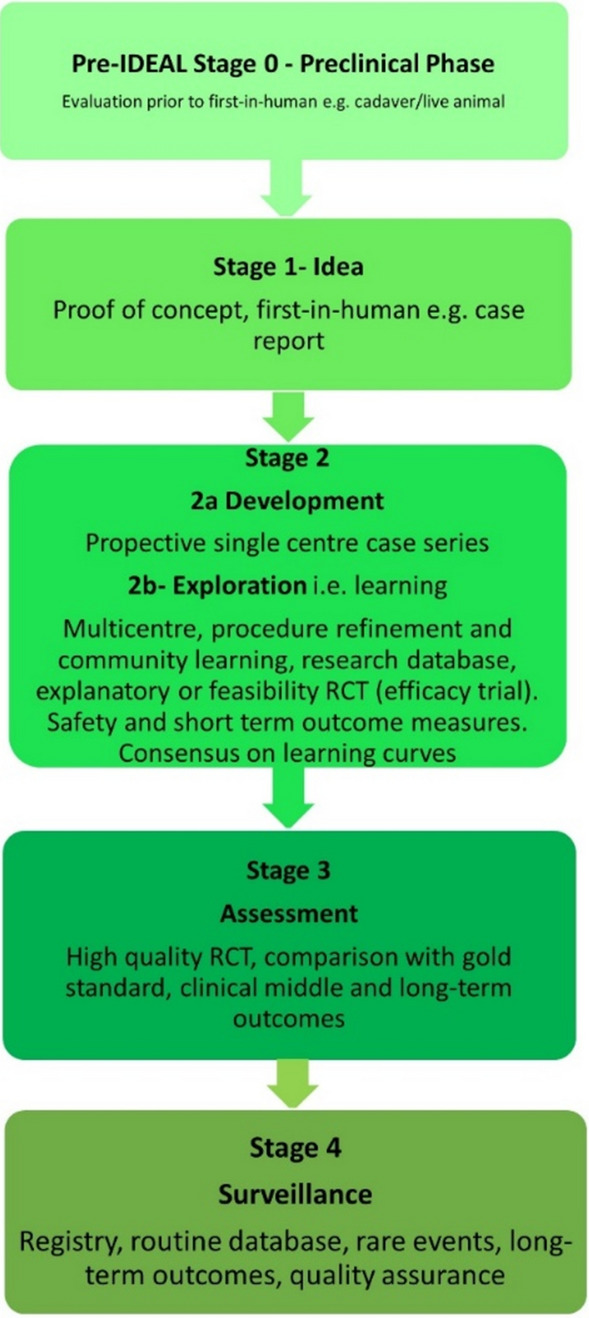
IDEAL Framework stages modified from the website

Information from the public domain was also collected and each company approached via email for virtual interview to discover more about the systems and quality check data collection. This was a structured hour-long interview with a template of questions used (Fig. 1), and the company was given an opportunity for a short presentation. Clinical data to aid the IDEAL stage of evaluation was identified through PubMed, Google and company websites. Data collection included: company, founding year, development and testing including pre-clinical/clinical trials, price, system and device descriptors, training and support available, and additional information distinguishing robots from their competitors. The IDEAL Framework (Fig. 2) was applied to assess the stage of evaluation for each system in the clinical setting. All companies who responded reviewed their respective data in this review as part of the quality assurance process. Results are accurate to the authors’ knowledge at the time of publication, however, may have changed or inaccuracies present, particularly in companies who have not responded.

## Results

A total of 36 robotic system platforms were identified for potential data extraction. Of these, 15 were excluded, 10 of which were endoluminal or natural orifice robotic systems and the other 5 were robotic devices rather than surgical systems. A total of 21 robotic platforms were scrutinised for data extraction and analysis, presented in full in Table 1, with accompanying images in Figs. 3, 4, 5, 6, 7, 8, 9, 10, 11, 12, 13, 14, 15, 16, 17, 18, 19, 20, 21, 22.

**Table 1:** Robotic systems description and evaluation

Robotic system	Origin and use	Price	Regulatory approval and indications	Device description	Training pathways	IDEAL Framework Stage and evidence
**da Vinci, Intuitive Surgical, Inc** [16].* Figure 3	USA, 1995 (Intuitive Surgical founded) Launched 1st generation in 2000 after FDA approval for general laparoscopic surgery Merged with ZEUS Robotic Surgical System 2003 **1st operation** 2000 **Systems in use as of 31/12/22:** 7,544 systems: 4,563 in the US, 1,388 in Europe, 1,234 in Asia and 359 in the R.O.W **Operations performed as of 31/12/22:** Estimated 1,875,000 during 2022, and over 10 million in total	Xi ~ $2 million with per procedure costs of roughly $2500 [17] X stated ~ $1.2 million in SSI Mantra technology differentiator brochure	**Approvals:** CE mark, FDA, Japanese Ministry of Health, Labour & Welfare (MHLW) 2012, China National Health Commissions 2018, UKCA Mark needed for GB market post June 2023 **Indications multiport:** General surgery, urology, gynaecology, thoracic, paediatrics, plastics, head and neck specialties **Indications for SP:** Urology, TORS, and expanding	Set up: Master slave with surgeon, patient and vision cart. st generation da Vinci Surgical System 2nd generation da Vinci S 3rd generation da Vinci Si 4th generation Xi, X and single port (SP) **Xi:** advanced instrumentation, vision, and features e.g. integrated table motion, setup automation allowing target anatomy and positioning guidance. Xi has a boom allowing multi-quadrant access **X**: cost-conscious options with the same arm architecture as Xi ability for advanced analytics **SP:** Flexible platform configuration, Single port 25mm cannula insertion holding three instruments and articulated 3D endoscope **Vision:** 3DHD **Instruments**: 8mm to 12mm, reusable (12 to 18 uses), has advanced energy and staplers **Additional information:** Firefly Fluorescence Imaging combined in the endoscope Additional robots include Ion Endoluminal system for minimally invasive biopsies Intuitive Hub System and My Intuitive app to record and track progress. Development of machine learning tools to help improve evaluation (launch stated hopefully 2023) Intuitive offers grants for fellowships and research and development	**Training**: Established modular training pathways TR100, TR200, TR300, TR400 and TR500 as in-hospital proctors. Includes training with patient cart assistance **Virtual reality**: Multiple validation studies of simulators [18] (dV-Trainer, da Vinci Skills Simulation from Mimic Technologies™ and da Vinci SimNow™), provide basic and procedure-specific simulation	Multiport—Stage 4 Single Port- Stage 3 **Supporting evidence:** > 34,000 peer reviewed clinical journals Multiple Studies, including RCTs, show long-term outcome data for multiport [19] Single port in urology systematic review 2021 showed safety [20] and shorter hospital stay/less pain with otherwise comparable outcomes vs multiport in a systematic review and meta-analysis [21, 22] da Vinci Application Programmer Interface via dVLogger recording device provide automated performance metrics which have been evaluated with concurrent [23]– [26], construct [27]– [29] and predictive [29]– [32] validity in some studies, with new research focusing on the application of AI
**KANGDUO ROBOT® Surgical System (KD-SR-01), Suzhou Kangduo Robot Co., Ltd** Figure 4	China, 2013 Collaborating with University Harbin Institute for Technology **Countries in use:** China **1st operation**: Pyeloplasty 7/8/2020 Currently 20 systems in use	**Price:** $1–1.4 million	**Approvals**: National Medicinal Products Administration (NMPA) of China Approval 2022 Approvals in Europe, Russia and Sri Lanka in progress **Indications:** Urology, gynaecology, general surgery, thoracics	**Set up:** Similarities to da Vinci: Surgeon, patient and vision cart Foot pedals have the same design as the da Vinci Cross laser positioning system and boom like Xi Filter tremor and motion scaling Differences: Only 3 arms and an open console **Instruments:** Fourteen 8mm instruments fenestrated grasper, double fenestrated grasper, tip-up double fenestrated grasper, Cadiere forceps, curved dissector, monopolar straight and curved scissors, large and small hook, Maryland bipolar forceps and large/small needle driver, fenestrated bipolar forceps, mini non-invasive round tip fenestrated Reusable up to 10 times **Ergonomics:** Seated, armrest **Vision**: 3DHD with glasses, compatible with most 10mm 3D endoscopes **Additional:** ICG and 4KHD Fluorescence Reconstruction and USS capability 5G remote surgery performed on a live animal successfully Future models SR1500 and SR2000 in development and will include AI capabilities	**Training:** Training centre in Beijing 1–2 days with animal operating **Virtual reality**: Co-designed with Simbionix, embedded within the surgeon console	Stage 3 **Supporting evidence:** Pre-IDEAL (Stage 0)- RCT on porcine models [33] Stage 1: First in human B/L pyeloplasty for horseshoe kidney [34] Stage 2a: Case reports on partial Nephrectomy [35, 36] Prospective single-centre study for pyeloplasty [37] Stage 2b: Prospective single arm studies for RARP [38], partial adrenalectomy [39], pyeloplasty comparing to da Vinci Si, da Vinci outperformed in operative time and time per stitch [40] Stage 3: RARP- KangDuo vs da Vinci Si, comparable short-term functional and oncological outcomes [41] 2 centre blinded RCT showing non-inferiority with da Vinci Si in RAPN for T1a renal tumours [42]
**Senhance® Surgical Robotic System, Asensus Surgical Inc** Figure 5	USA, 2013–2016 developed and known as Telelap ALF-X. 2016 Launched with TransEnterix which became Asensus Surgical in 2021 **1st Operation:** Hysterectomy, Rome, Italy [43] **Use:** 10,000 + operations worldwide 39 clinical sites US, Europe and Asia 6 global training centres 250 + active surgeon users	$1–1.2 million with per procedure costs ~ $1000 [17] Benign hysterectomy: Procedure cost $1,393 da Vinci, $559 Senhance, laparoscopic $498 [44]	**Approvals:** FDA and CE mark 2017 MHLW 2019 Further CE mark, FDA, MHLW approval for machine vision and augmented intelligence 2023 **Indications:** Urology, General Surgery, Thoracic, Gynaecology, Paediatrics	**Set up:** Open console from the surgeon’s cockpit Modular system: Four manipulator arms (Japan and CE Markets only) are standalone units with digital fulcrum point; therefore, no docking to minimise trauma and increase workspace at the trocar Hand controller as a laparoscopic instrument **Ergonomics:** Seated in the surgeon’s cockpit, adjustable settings **Instruments:** Reusable, most are laparoscopic (non-articulated) 5mm bipolar grasper and needle driver are articulated Hand/instrument movements are traditionally laparoscopic Haptic feedback- an enhanced sense of force pressure and tension 3mm, 5mm and 10mm instruments. 70 + types, including advanced ultrasonic device **Vision:** 3DHD with glasses Eye tracking device, supplied by Tobii, is activated by the surgeon and moves the camera to the point of vision **Portability:** Easily portable due to bedside unit arms i.e. modular units **Additional:** Open platform architecture to be compatible with 3DHD and HD fluorescence systems, negating the need for software updates, as well as electrocautery units currently in the hospital, improving the economic feasibility Machine vision/augmented intelligence- Intelligent Surgical Unit (ISU). Current capabilities include the automatic following of the tip of the instrument, smart zoom if you have a degreed scope keeping the image centred on relevant operative anatomy, and real-time, point-to-point and contour measurement allowing for example mesh sizing. In development: 3D mapping, instrument recognition, organ/critical structure detection	**Training:** Whole team training 2 day dry and 1 day wet lab in Milan or Amsterdam. Scheduled case the week after for surgical proctor to attend the surgeon’s hospital, with a clinical specialist to support until fully independent	Stage 3 **Supporting evidence:** Clinical evidence, 80 + reviewed publications, for 8 years, multicentre, multispecialty observational studies including from The TransEnterix European Patient Registry for Robotic-Assisted Laparoscopic Procedures in Urology, Abdominal, Thoracic, and Gynaecologic Surgery ("TRUST"). It is the largest multi-specialty robot-assisted laparoscopy registry. 2500 + patients enrolled Safety and efficacy with low conversion and low complication rate from publications [45]
hinotori™ Surgical Robot System, Medicaroid Corporation** Figure 6	Japan, 2013 Jointly established by Kawasaki Heavy Industries Ltd., and Sysmex Corporation **1st operation:** December 2020	Not publicly available, states less than the equivalent 270 million yen of da Vinci Xi	**Approvals:** Japanese Ministry of Health Labour & Welfare (MHLW) approval in August 2020 **Indication and use in:** Urology, General Surgery- gastrectomy 2022 [46], colorectal [47] and gynaecology [48]	**Set up: “**Operation unit” with 4 arms, 3 instruments and 1 endoscope holder on an “arm base” boom “Surgical cockpit” has a 3D viewer, touch panel, hand control grips and foot unit similar to Intuitive’s design Compact and safety design to minimise collisions and detect arm/port movement errors (Cubic-S), plus a vibration filtration The pivot point position of the trocar on the patient’s body wall is monitored by Cubic-S. The software allows more workspace around the trocar as the arms do not dock to the trocar, therefore, there is a reduced risk of clashing with the patient or robotic arms Robotic function can be enabled at the arm, operation cart panel or when the surgeon looks into the viewer **Ergonomics:** Adjustable settings at the surgical cockpit **Vision:** 3D HD **Instruments:** Sterilisation with autoclave. Can be used up to 10 times **Portability:** Compact, easily transferable between ORs **Additional:** Designed to be 5G compatible, anticipating telesurgery/remote operating, with an initial animal cadaver feasibility study showing safety in 2022 [49]	**Training:** The whole healthcare team at Kobe University Hospital International Clinical Cancer Research Centre	Stage 2b **Supporting evidence:** Single centre prospective study 30 patients for RAPN [50] and a multicentre study of 30 patients for RARP [51]
Hugo™ RAS System, Medtronic* Figure 7	USA, Launched 2021 **Countries in use:** Australia, Belgium, Brazil, Canada, Chile, Denmark, Finland, France, Germany, India, Israel, Italy, Japan, Netherlands, Panama, Portugal, Spain, Sweden, Taiwan, United Kingdom **1st operation**: RARP, Chile 2021 [52]	**Price:** Stated to be 20–25% cheaper than da Vinci Xi	**Approvals**: CE mark 2021; Health Canada License; MHLW Japan; Not FDA approved **Indications:** Urology, gynaecology, general surgery	**Set up:** Modular, multi-quadrant platform Surgeon console: Open console, pistol grip controllers, with motion scaling variability, compatibility with the Hugo task simulator. Pedals control the arms, energy supply (monopolar, bipolar), master clutch, camera control and arm switching Arm cart: Three and four arm configurations, alternatively one arm can be used to assist in laparoscopic procedures Hugo Tower: Touch screen monitor and central processing unit for the robotic system featuring KARL STORZ technologies, Valleylab™ FT10 energy platform and TouchSurgery™ Enterprise video management and analytics platform **Instruments:** Standard instruments and monopolar/bipolar. No advanced energy or stapling devices **Ergonomics:** Seated at surgeon console, adjustable **Vision**: 3DHD with glasses, endoscope on any arm. Head tracking system which will disable control of the instruments if the surgeon is not looking at the monitor **Portability:** The modular system is easily portable **Additional:** Cloud-based surgical video capture option in Touch Surgery record procedures and gather data for viewing and analysis	**Training:** HUGO ASCEND Training Pathway. Modular training pathway with Technical Training (Medtronic Staff), Procedural Experience (by surgeons), Coaching (Surgeon to surgeon proctoring) Other: Onsite on demand support from engineers **Virtual reality:** Hugo™ task simulator, like da Vinci backpack simulators, attaches to the surgeon console	Stage 2b **Supporting evidence:** Stage 0: Report on left and right colectomy in cadavers Jan 2023 [53] Stage 1: Multiple reports of first in-human cases for urology, gynaecology 2022 [52] and general surgery [54, 55] Stage 2a: Case series in urology [56, 57] Stage 2b: 112 patients undergoing RARP [58], 60 patients sacrocolpopexy [59]
MicroHand S Surgical Robot, Shandong WEGO Surgery Robot Co., Ltd. & ZCo Design Co., Ltd.** Figure 8	China, 2013 Developed by Central South University and Tianjin University **1st operation in trial**: 2014	Not publicly available	**Approvals:** NMPA approval 2021 (in certain general surgery procedures) [60]	**Set up:** “Doctor’s console” is open with an armrest, and finger grip hand controller Patient console has 3 robotic arms **Vision:** Vision console. 3DHD view **Ports**: 10mm trocars for the robotic arms **Additional:** Incorporated 5G to allow remote operating, performing a radical cystectomy [61]		Stage 2b **Supporting evidence:** Single centre prospective centres: - Lap vs robotic right hemi 22 patients, robotic outperformed [62] - Versus da Vinci 45 patients undergoing sigmoid colectomy. Microhand shorter length of stay and improved hospital costs [63] - Robotic TME vs. da Vinci Si, no difference found in patients’ genitourinary function; both outperformed lap [64] - Other: sleeve gastrectomy (n = 7) [65] and a dry lab exercise showing construct validity of kinematic data on the MicroHand S robot
**Revo-I, Meerecompany Inc** Figure 9	Republic of Korea, 2007 **1**^**st**^** human trials:** Cholecystectomies 2016 [66] **1**^**st**^** operation (after approvals):** RARP 2018 **In use:** USA, Asia including Uzbekistan, Japan, Europe and R.O.W **Number of ops worldwide**: 200 + by September 2021	Not publicly available but states a reduced price to be available in more countries	**Approvals:** Ministry of Food and Drug Safety (MFDS) Approval 2018 **Indications:** General Surgery, gynaecology, urology, ENT	**Set up:** Similar to da Vinci Si Master console, vision and patient cart Surgeon console is closed **Ergonomics:** Seated, adjustable **Instruments:** 13 instrument types, short and long, 26 in total. Monopolar, bipolar, scissors, forceps, needle holders and clip appliers **Vision:** 3DHD	**Training:** Modular training pathway: online, dry/wet lab, procedural skills then robotic skill assessment Supports with logistics, technical, marketing, open R&D, Revo Clinical Support Consultant **Virtual reality:** Revo-Sim with three modules- basic to advanced including procedural. 30 total tasks	Stage 2b **Supporting evidence:** Stage 1: First case in human case reports including prostatectomy and pancreatectomy [67] Stage 2a/2b Prospective cohort study 17 patients undergoing RARP [68] Prospective cohort study 15 patients undergoing cholecystectomy [66] Stage 2b: Equivalent short term functional and oncological outcomes in propensity score analysis of 33 in each cohort Revo-I vs da Vinci Si [69]
**Toumai® Laparoscopic Surgical Robot (MicroPort® Toumai®)** Shanghai MicroPort MedBot (Group) Co., Ltd. (MicroPort® MedBot®)** [70] Figure 10	China, 2014 Design completed 2018 **Use:** Over 30 Chinese centres	Not publicly available	**Approvals:** National Medical Products Administration (NMPA), 2022 for Urology **Indications:** Urology, gynaecology, general surgery, thoracics [71]	**Set up:** Similar to da Vinci Xi with 4 arms on a boom. Tremor filtration Surgeon, vision and patient console **Vision:** 3DHD **Additional:** Incorporated 5G for remote operating [71]. Stating world’s longest range performed (> 5000km) in June 2022 Toumai ® 2^nd^ generation awaiting NMPA approval Toumai ® single arm commencing enrolment for clinical trials, completed 1^st^ in human Other product including natural orifice and bronchoscopy developed	**Training:** 400 + senior surgeons trained, ~ 1500 training procedures complete	Stage 2b **Supporting evidence:** Clinical validation study of 1st generation, > 300 procedures in urology, gastrointestinal and gynaecology Completed clinical trials for 2nd generation in gynaecology, thoracics and general surgery undergoing registration application phase
**Versius®, Cambridge Medical Robotics (CMR) Surgical** Figure 11	UK, 2014 **1**^**st**^** Operation:** 2019 **Use:** Over 100 systems installed with over 5000 cases performed (November 2022)	Not publicly available Flexible, bespoke contracts, including subscription/lease/capital purchase models	**Approvals:** CE mark March 2019 **Indications:** Adult General surgery, gynaecology, urology, thoracics **Contraindications:** Paediatrics, Surgery relating to the circulatory or nervous system, radical hysterectomies with early-stage cervical cancer	**Set up:** Open console, modular system Hand controller likened to a split video game controller **Ergonomics:** Adjustable settings, armrest, seated or standing **Instruments:** 6 fully articulated instruments **Vision:** 3DHD with glasses **Portability:** Footprint of each bedside unit arm: height 1425 mm/width 380 mm/depth 380 mm, height adjustable, neutral/sleep position is smaller than most average human height. Allows access to the patient **Additional:** “Soft” or “collaborative” robotics, doesn’t use a pully system like other robotic platforms, it has motors with torque sensors. This allows external force applied to the arm as a safety feature	**Training:** Modular, whole team training pathway including e-learning (80% pass), dry and wet lab procedure run through Preceptor present to check happy with the robot at the clinical site Additional training offered **Virtual reality**: Versius Trainer	Stage 2b **Supporting evidence:** Stage 1/2a: First in human studies of 30 cases included cholecystectomy, appendicectomy, diagnostic laparoscopy, and gynaecological operations [72] Stage 2b: Prospective observational study 32 participants in colorectal [73] Other feasibility and pre-clinical studies are available on their website
MP1000 and SP1000 robots, Shenzen Edge Medical Robotics Co.** Figure 12 **Note:** Unable to view the website, information has been taken from publications, web reports and draft material which may not accurately reflect the company or the robotic device	China, 2017 **Operations in trial:** RARP 2021 Nephrectomy 2021 Hysterectomy 2021 Oophorectomy 2021 Distal gastrectomy 2022 Left hemicolectomy 2022 Pneumonectomy 2022	Not publicly available	**Approvals:** Multiport system MP1000 approved in urological surgery by Chinese National Medical Products Administration (NMPA) in December 2022 [74] Single Port (SP1000) under preclinical trials [74] Likely NMPA approvals in 2023 for other specialties and SP1000 **Indications:** General surgery urology, gynaecology and thoracics	**Set up:** Multiport system MP1000 Standard master–slave set-up control with a surgeon, vision and patient cart with a 4-arm structure [75, 76] Single port system SP1000 Same setup, 3 articulating arms with 7 DOF and an endoscope with 5 DOF. Same surgeon console for both robots Photos suggest almost identical setup and controls to da Vinci Xi and da Vinci SP In comparison to da Vinci SP with 4 DOF in endoscope, Edge Medical states 5 DOF **Instruments:** 30 instruments, bipolar and monopolar Developing Edge Robotic Ultrasonic Shears and Edge Robotic Stapler (stating this may not be successful) [60] **Vision:** 3DHD **Additional:** Developing Edge Bronchoscope Robot		Stage 2a **Supporting evidence:** MP1000 Stage 2a (pending 2b): 1st clinical trial in 2021 [60] Ongoing/finishing gynaecology clinical trial [60] Initiated clinical trials and aimed to finish recruitment November 2022 in general surgery and thoracics SP1000 Stage 0: Pre-clinical safety and feasibility in live porcine for nephrectomy [77] and taTME [78] Stage 1/2a: SP1000 completed the operations of its first clinical trial in gynaecology [60]. A webpage report (May 2022) stated it has performed an in human ovarian cyst removal [79]
**Dexter, Distalmotion®** Figure 13	Switzerland, 2012 Countries in use: Approval for use in European countries at this time 1^st^ operation: Rectopexy 2021, Gynaecology 2022, RARP 2022 [80]	Not publicly available	Approval: CE mark 2020 Indications: Urology, gynaecology, general surgery	Set up: Open console, modular system with two instrument arms and a robotic endoscope holder On-demand laparoscopic and robotic platforms as the surgeon is sterile, allowing easy switching between the bedside and surgeon console. Use of traditional laparoscopic port positions The hand controller is a pistol grip with finger and thumb paddles to open/close Two foot pedals for clutch and camera Instruments: Wristed, single-use, provided by Dexter. Five types including Johan, Maryland, monopolar hook, monopolar scissors, and needle holder. Open platform i.e., use of existing hospital laparoscopic tower and endoscope (if 3D) Ergonomics: Fully adjustable to sit or stand Vision: Open platform, 3D with glasses, imaging device agnostic which allows fluorescence imaging to be kept up-to-date as technology advances Portability: Motorised, portable to other ORs	Training: The Dexter Academy™. Modular training; didactic online off and on-site training including dry, wet (cadaver/live animal) Dedicated clinical application specialist per site	Stage 2a **Supporting evidence:** Stage 1: nephrectomy [81], pelvic organ prolapse [82] Stage 2a: Case series of 30 inguinal hernia repairs [83] and of the first 10 prostatectomies Pending 2b: Recruitment for prospective clinical study ongoing ClinicalTrials.gov Identifier NCT05537727
**avatera® system, avateramedical GmbH** Figure 14	Germany, 2011 **1**^**st**^** operation** RARP, 2022 **Countries in use:** Germany, Denmark, Greece, France, Hungary and others	Not publicly available ~ €1 million ($1.1) in 2019 [84]	**Approval:** CE mark 2019 **Indications:** For use with patients who are eligible for laparoscopic surgical procedures according to the surgeon’s assessment and decision. It has been validated for urologic laparoscopic surgery such as prostatectomy, cystectomy, lymph node removal, ureter surgery and (partial) nephrectomy and gynaecological laparoscopic surgery such as hysterectomy, myomectomy and resection of endometriosis	**Set up**: 4-arm robotic unit, surgeon control unit and optional electrosurgery cart. Two joystick hand controllers with straps and finger clutches Six-foot pedals for electrosurgery, camera and instrument switch Eyepiece designed to leave ears and mouth uncovered **Instruments:** Single use (except reusable endoscope), 5mm. Only bipolar available currently **Ergonomics**: Built-in ergonomic chair, adjustable settings, stereoscopic eyepiece with headrest, ears “free” for communicating **Vision:** 3D stereoscopic magnified view QXGA resolution (2048 × 1536px), overlay information on instruments, electrosurgery usage, statuses, and alarms **Portability:** Easily moved by one person between rooms through standard doors **Additional highlights**: Next generation in development The current system is very quiet within the OR due to no external fans	Modular training is available for the whole team with VR, Dry and wet (animal) lab Proctors available for onsite training with performance evaluation **Virtual reality**: In system simulation available	Stage 1 **Supporting evidence:** Stage 0: Feasibility of robotic bilateral nephrectomy and radical cystectomy live porcine models [85, 86] Pending 2b: A current multicentre trial is running and registered on ISRCTN45854742
Mantra Surgical Robotic System, M/S. Sudhir, Srivastava Innovations (SSI) PVT. Ltd* Figure 15	India, 2016 **1**^**st**^** Operation:** July 2022 **In use:** India, Sri Lanka, Nepal, Bangladesh and Indonesia	**Price:** $625,000	**Approvals:** CDSCO, India Filing for CE mid 2023 **Indications:** Cardiothoracic, Urology, General Surgery, Head and Neck, Gynaecology	**Set up:** Open console, modular 3–5 arm system, foot pedals with arm switch, clutch, camera control and electrocautery Vision cart has live streaming and recording capability for teletraining/telementoring **Ergonomics:** Seated, adjustable **Instruments:** 30 + 9mm instruments (SSI MUDRA™) including monopolar, bipolar, and clip appliers. No advanced energy yet Reusable with autoclave sterilisation Developing other instruments **Vision:** 3DHD, head tracking camera safety feature, articulating endoscope **Portability:** Modular with bedside units more easily portable, stowed dimensions are 610mm x 450mm x 1640mm **Additional:** Augmented reality intraoperative 3D holographic representation of patient’s anatomy with accurate MRI/CT scans	**Training:** Currently only provided in India **Virtual reality:** SSI Mantra Virtual Reality Simulator	Stage 1 **Supporting evidence**: Nil published currently, but in clinical use
MIRA Surgical System, Virtual Incision Corp.* Figure 16	USA, 2006 **1**^**st**^** operation:** Right hemicolectomy August 2021	N/A currently not for sale	**Approvals:** The MIRA Surgical System is an Investigational Device and is not currently available for sale **Indications:** N/A	**Minibot and camera:** Single port through ~ 2.5cm incision MIRA features two robotic arms and an articulating high-definition camera. Motors are located inside the arms, with the goal of achieving triangulation at sufficient strength and dexterity, even for complex procedures like colon resection The miniaturised design reduces the external footprint, potentially eliminating the need for dedicated OR space. It also enables multi-quadrant access with no external arm collisions **Surgeon console:** Open console design Hand controls have open-close paddles and sensors to detect the user, with a clutch function in the left hand and a camera function in the right Foot pedals (clutch, camera, left hand bipolar, right hand monopolar) **Ergonomics:** Seated, open console maintaining peripheral vision of the operating room **Vision**: Full HD resolution in real-time on the main display **Portability**: MIRA’s miniaturised design is portable and aims to be easily set up in any operating room in a matter of minutes, eliminating the need for dedicated OR space and long turnover times. The minibot and camera are sterilised between cases like other instruments and stored on the shelf in a sterile tray. Just before the procedure, it is mounted on the patient table with no need for draping or docking **Haptic feedback**: A haptic indicator detects when instruments are out of range **Additional:** Surgeon App (MIRA IQ) currently under development	**Training:** Extensive training will be provided by the company **Virtual reality:** Simulator (MIRA Sim) currently under development	Stage 1 **Supporting evidence:** Stage 0: Pre-clinical animal studies [87, 88] Stage 1: First in human reported on their website but no clinical trials published Pending Stage 2b: At 3 centres currently undergoing trial. ClinicalTrials.gov Identifier: NCT04703829
Shurui Robot, Beijing Shurui Technology Co., Ltd** Figure 17 [106]	China, 2014	Not publicly available	**Approvals:** 2020 Passed special review procedure of Innovative Medical Devices of the State Food and Drug Administration No other information available **Indications:** Urology, gynaecology, general surgery, and thoracics	**Set up:** Operating trolley: Single port, 3 instruments and articulating endoscope. Differs from other single port as it has 1 arm per instrument feeding into 1 operating channel Main control trolley: Surgeon console with stereoscopic viewer Hand controller with digit grips similar to da Vinci Multi quadrant access External positioning arm remains motionless, increasing safety and reducing collision risks **Ergonomics:** Surgeon seated at main control trolley **Instruments:** Inserted on to separate arm that will then feed into common channel through the patient wall **Vision:** 3DHD		Stage 1 **Supporting evidence:** Stage 1: First domestic SP for gynaecology, RARP 2021 [89] partial nephrectomy 2021 [90], sigmoid colectomy in 2022 [91]. World first extra-peritoneal adrenalectomy 2021 [92]
Bitrack System, RobSurgical** [93] Figure 18	Spain, 2012 2014 1^st^ animal model operations	N/A currently not for sale Anticipated to be cheaper due to open platform	**Approvals:** No International Standards Organisation (ISO) 13,485 certification **Intended indications:** Urology, gynaecology, general surgery	**Set up:** Robot: 4 arms, extend from a column, generic trocars with general locations, smaller footprint. 2 passive joints avoids forces at the fulcrum point on the patient to reduce trauma, reducing clashing risk Surgeon console: Open console **Ergonomics:** Seated, adjustable with an arm rest **Instruments:** 8mm, single use, 7 DOF, monopolar and bipolar **Vision**: 3D **Additional:** Intended for hybrid laparoscopy and robotic procedures Open platform i.e. generic trocars, 3D screen, electrosurgical unit, and trocar location Developed AI systems: - Respiratory compensation system, - Intelligence Laparoscopic Navigation system	N/A	Stage 0 **Supporting evidence:** More than 30 in-vivo procedures performed
Carina™, Ronovo Surgical** Figure 19	China, 2021	N/A currently not for sale	**Approvals:** No	**Set up:** Modular system, 3–4 arms with a smaller footprint **Vision:** 3DHD	N/A	Stage 0 **Supporting evidence:** Animal and cadaver lab operations [94]
Enos™, Titan Medical Inc.* Figure 20	Canada, 2020 Rebranded from Single Port Orifice Robotic Technology -SPORT Expected human trials 2023 Expected product launch 2025 [95] Definitive agreement for collaboration with Medtronic confirmed for development of robotic technology [96]	N/A currently not for sale	**Approvals:** No IDE application data expected summer 2023	**Set up:** Open console, single port 25mm insertion tube with three articulating arms and camera Arms multiarticulated allowing four quadrant access, without external moving parts **Instruments:** Can be sterilised and reused, ten tip types that can be loaded and unloaded through insertion tube including monopolar hook, hunter and Maryland bipolar dissectors, needle driver, suture cut, tenaculum, fenestrated and laparoscopic clinch effectors **Ergonomics:** Seated, adjustable workstation **Vision**: 3DHD vision **Portability**: States smaller footprint, minimal cable management	**Virtual reality:** Integrated simulation software	Stage 0 **Supporting evidence:** Pre-clinical studies on pigs and cadaver [97] Website states > 80 pre-clinical lab procedures
Ottava, Johnson & Johnson**	USA Acquisition of Verb Surgical in 2019 [98] Announced in 2020	N/A currently not for sale	**Approvals:** N/A Possibly CE mark/FDA approval 2026 [98]	**Set up:** 6 arms to provide more control and flexibility in surgery which are integrated into the operating table. This zero-footprint design is to enable patient access, increase space in operating room and improve workflow – however there is scepticism regarding the six arms as this increases risk of clashes or entanglements Plans to combine with Auris’ Monarch robotic surgical platform to access and treat challenging anatomy in a minimally invasive way	N/A	Stage 0
**MiroSurge, Institute of Robotics and Mechatronics at the German Aerospace Centre (DLR)** Figure 21	Germany, 2017 Launched MIRO Innovation lab launched	N/A not for sale. Use in non-commercial/research purposes only Of note technological components of the MIRO robot were licensed to Medtronic in 2013 and utilised in HUGO™ RAS-System [99]	**Approvals**: No	**Set up:** MiroSurge is the entire telemanipulation modular system Each robotic arm is called MIRO Open console with 3D screen and glasses Hand controller and clutch foot pedal **Ergonomics**: Seated with arm rest **Instruments:** Currently use can support water jet for wound debridement, neurosurgery, US guided application, and robotic-assisted laparoscopy in development. Instruments are called MICA include forceps scissors, needle holder, palpation tip with a miniaturised sensor for haptic feedback). Sigma.7 provides artificial haptic feedback for system limitations e.g. joint limitation, collision avoidance **Vision:** 3D HD Storz **Portability:** Low weight of 10kg, accommodating 3kg payload, which can be mounted on side rails, walls, ceilings	N/A	Stage 0 – Note it will never go to in-human
Vicarious Surgical Inc** [100] Figure 22	USA, 2014	“Low cost of ownership”	**Approvals:** No, aiming for FDA clearance in 2024 Won the FDA Breakthrough Device Designation **Indications:** Targeting abdominal procedures particularly ventral hernia	**Set up:** Surgeon console, seated, arm rest, with cautery foot pedals and a 3D screen Single port incision through 1.2–1.8cm **Ergonomics:** Adjustable seating at the surgeon’s console **Instruments:** 2 working arms with 9 DOF, cantered pivoting inside the abdomen designed for the surgeon to work from any direction through any entry point **Vision:** 3DHD **Portability:** Patient and surgeon cart fit through a 34-inch (standard) doorway. Designed for easy setup, breakdown and storage	**Virtual reality:** Yes, with a VR headset	Stage 0 **Supporting evidence:** Stated performed well in cadaveric testing

Companies who underwent virtual interviews have no asterisk

*Companies who replied via email

**Companies who did not respond and information was gathered purely from the public domain

**Fig. 3:**
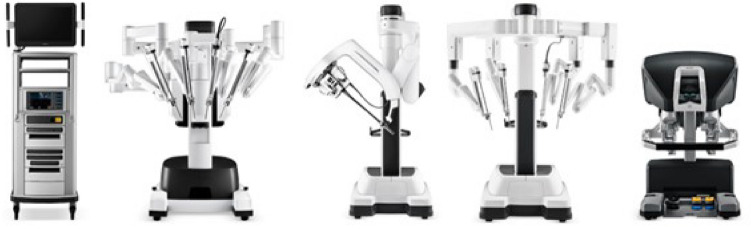
da Vinci 4th generation robots [101]

**Fig. 4:**
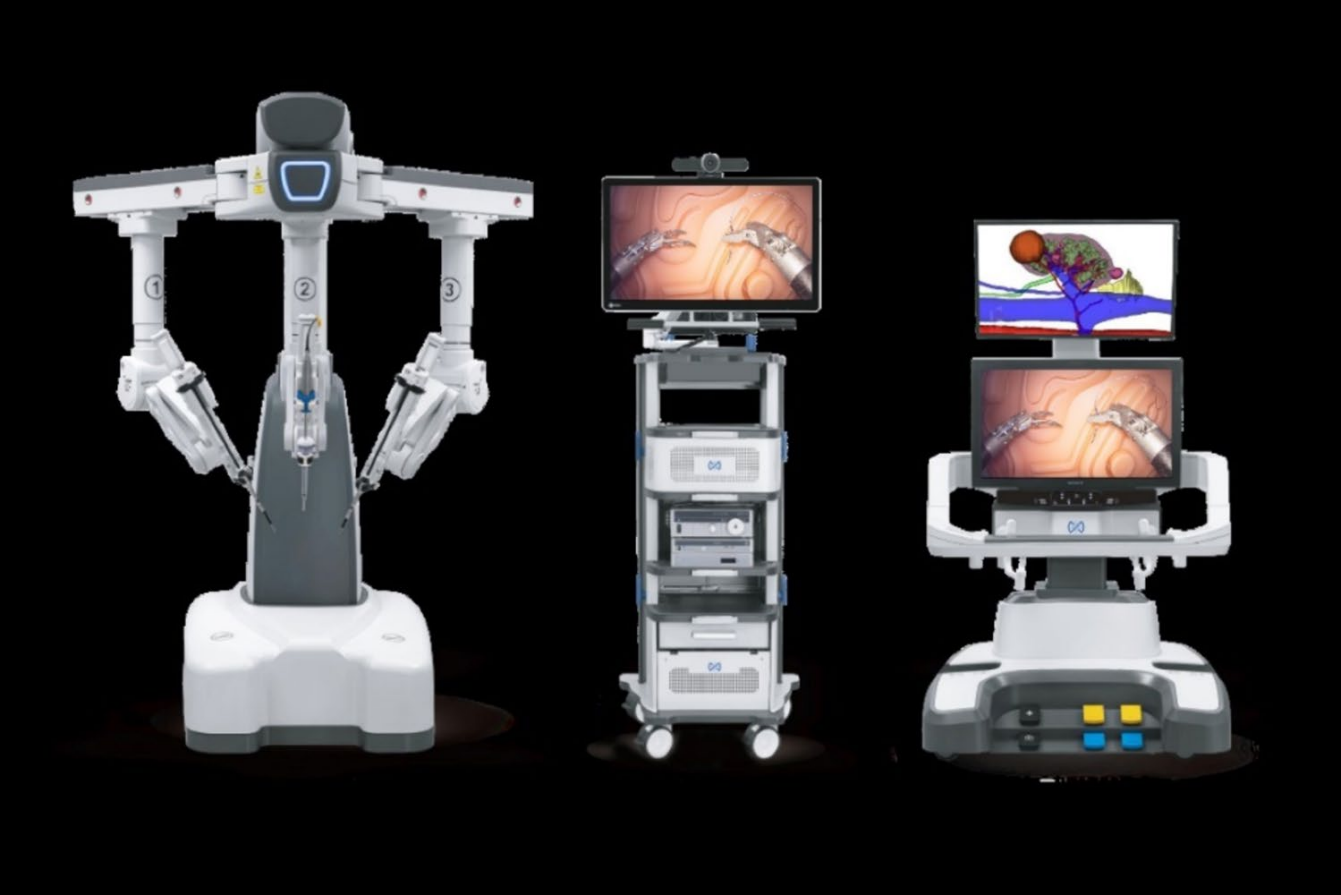
KANDUO Robot® Surgical System provided by and permission from Suzhuo Kangduo Robot Co., Ltd

**Fig. 5:**
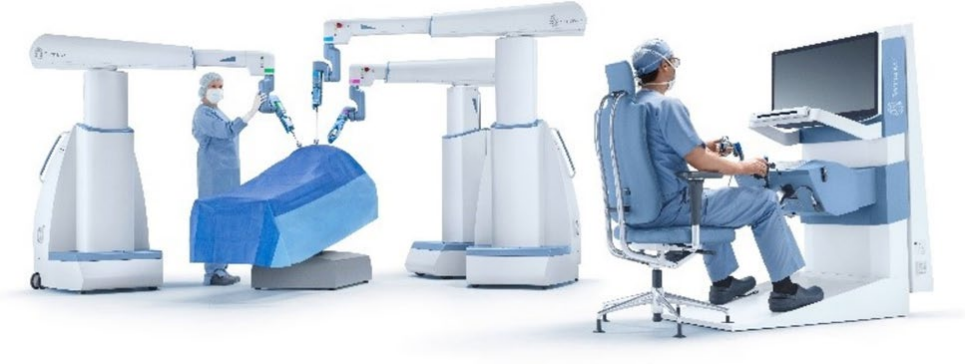
The Senhance® Surgical System, Asensus Surgical. Provided by and with permission from the company

**Fig. 6:**
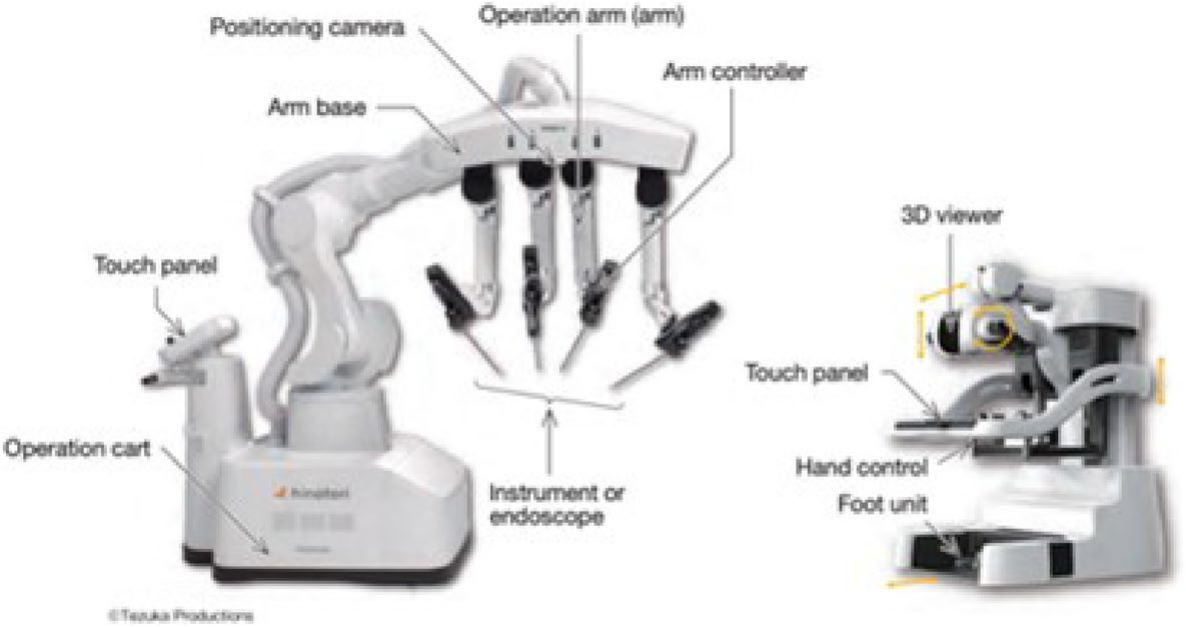
Hinotori Surgical Robot, Medicaroid Corporation [102]

**Fig. 7:**
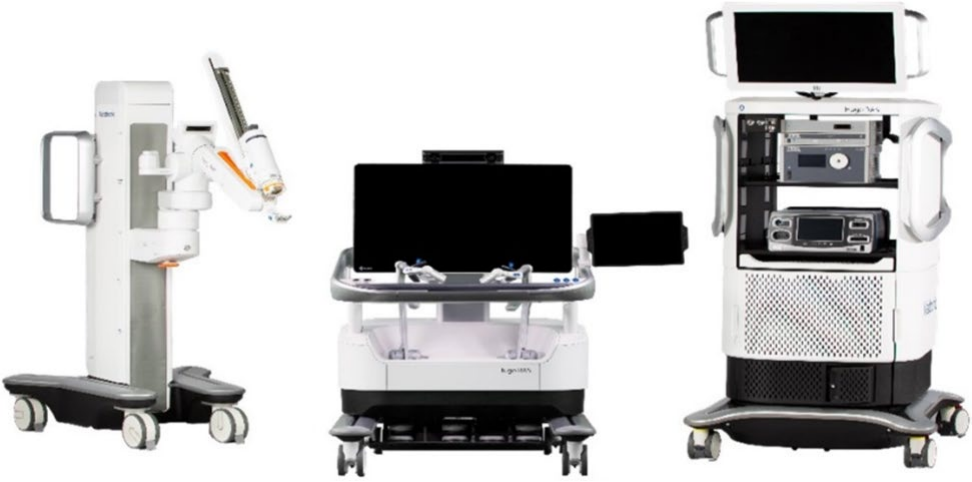
Hugo™ RAS system, Medtonic. Photos from media kit and with permission

**Fig. 8:**
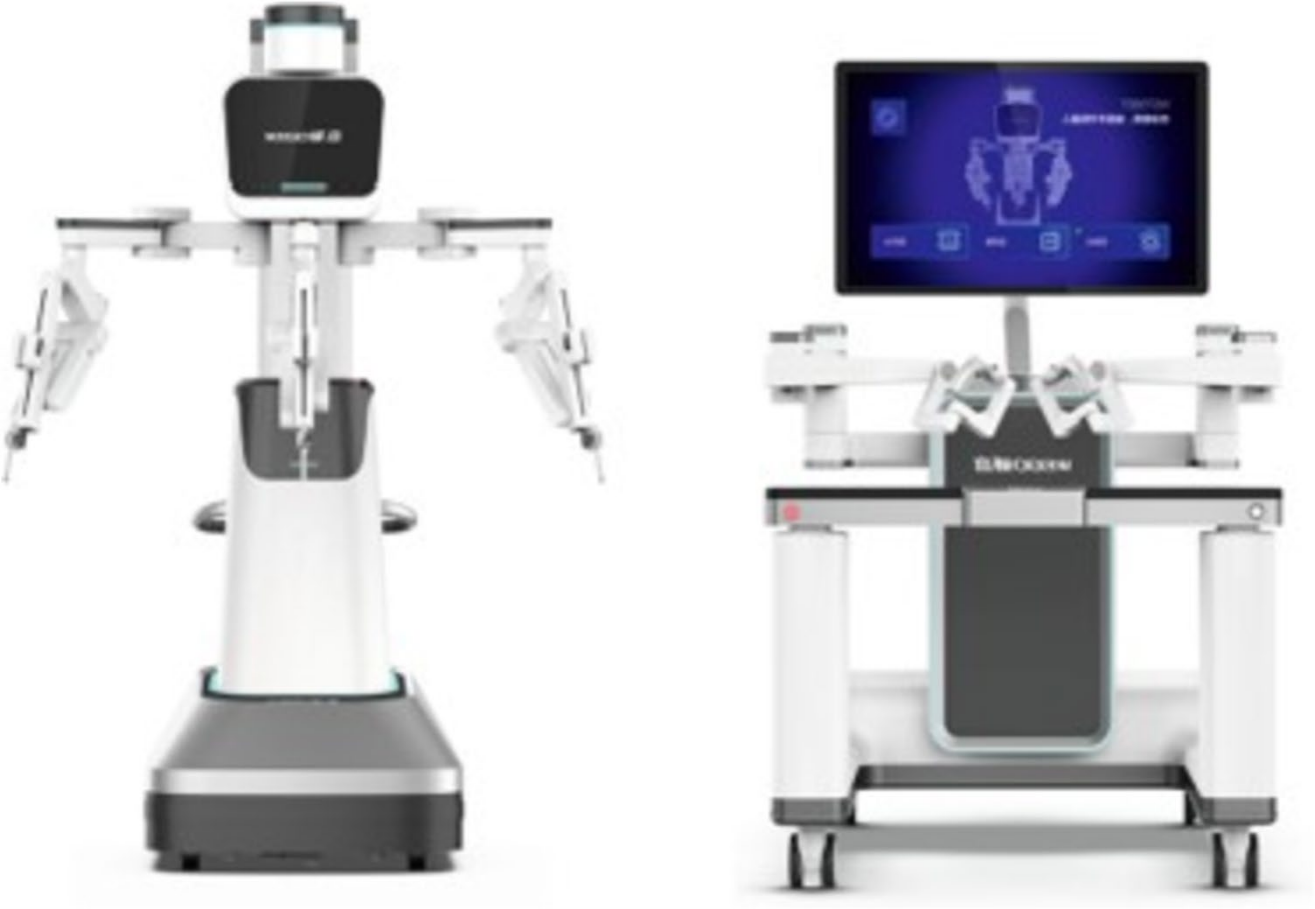
WEGO MicroHand S Surgical Robot System [103]

**Fig. 9:**
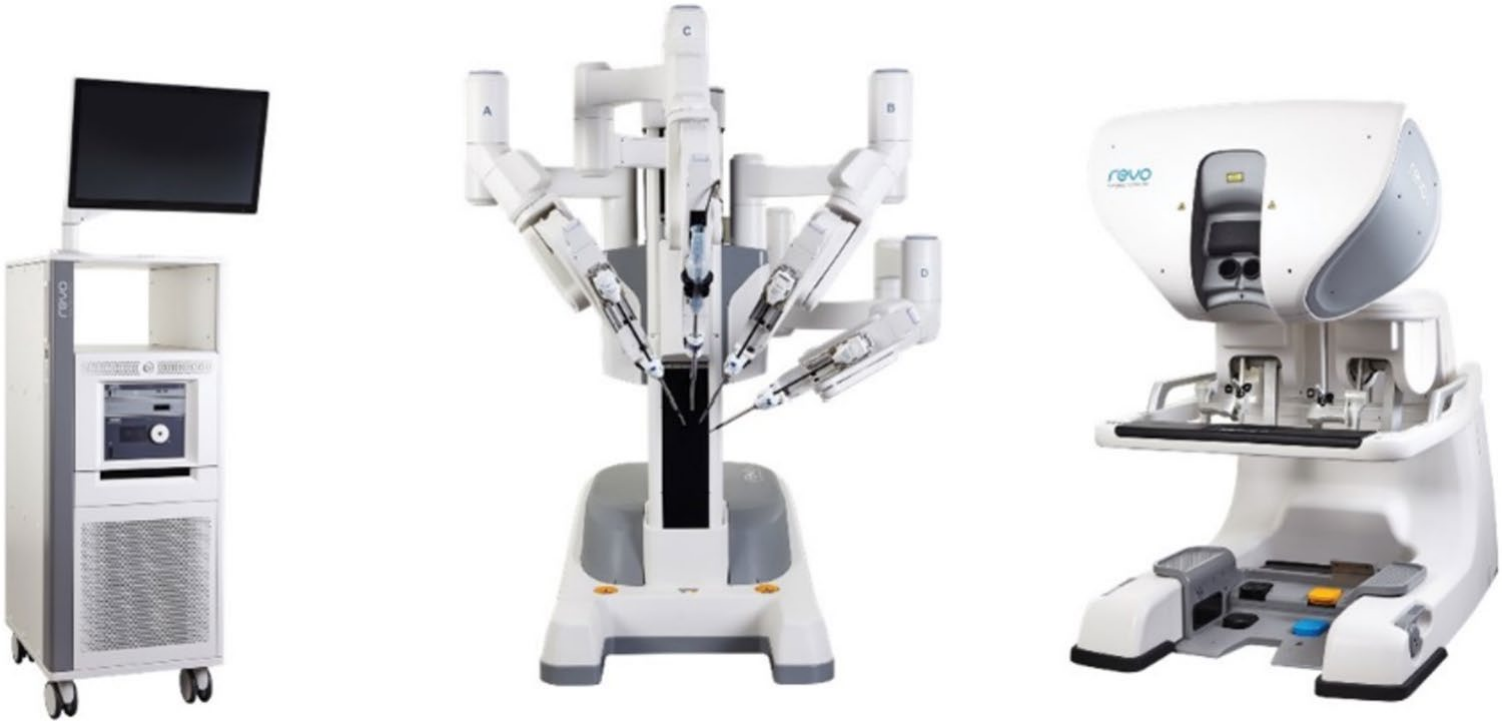
Revo-I system, Meerecompany Inc. Downloaded from the website media kit

**Fig. 10:**
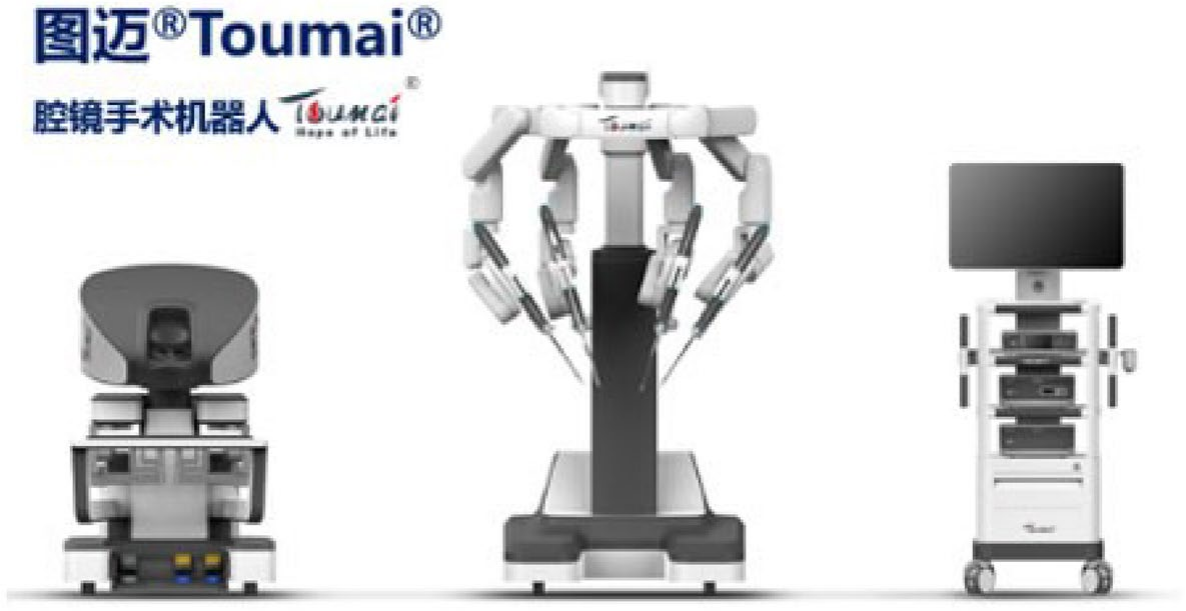
Toumai Laparoscopic Surgical Robot, MEDBOT [104]

**Fig. 11:**
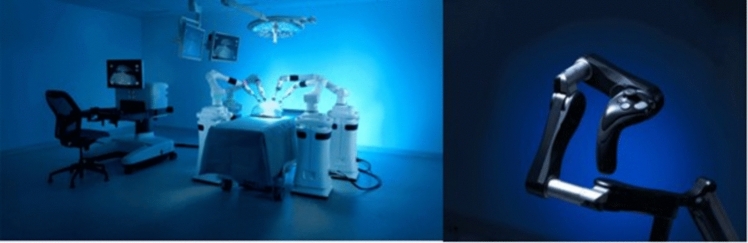
Versius Surgical Robotic System, provided by and permission from CMT (left picture- surgeon console and modular bed side units, right picture- hand controller) [105]

**Fig. 12:**
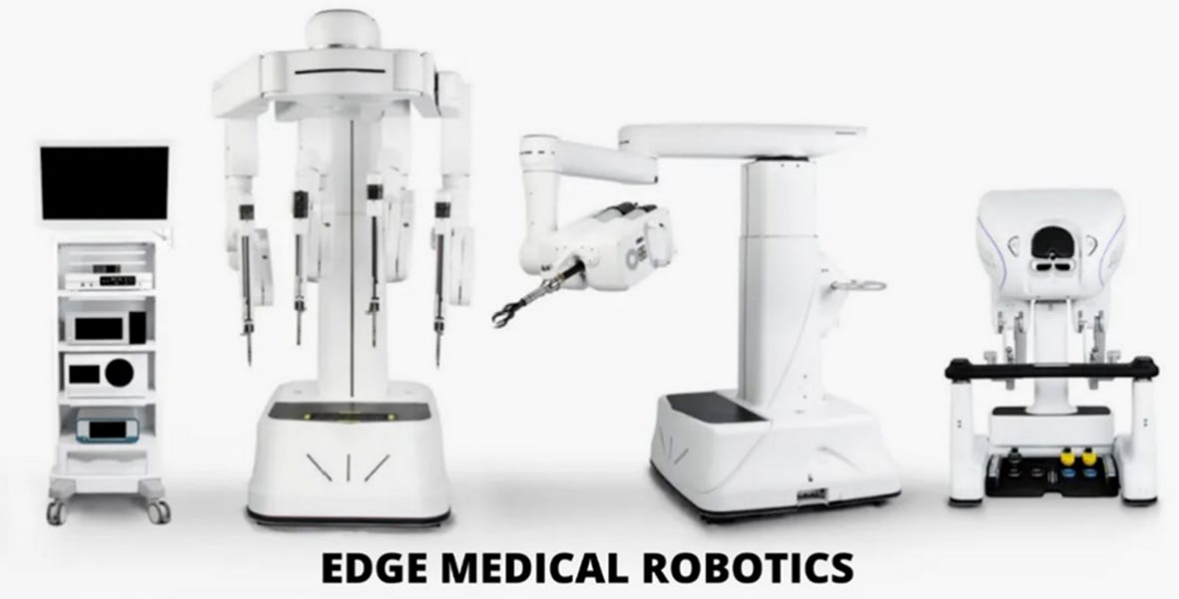
MP1000 and SP1000 robots, Shenzen Edge Medical Robotics Co [76]

**Fig. 13:**
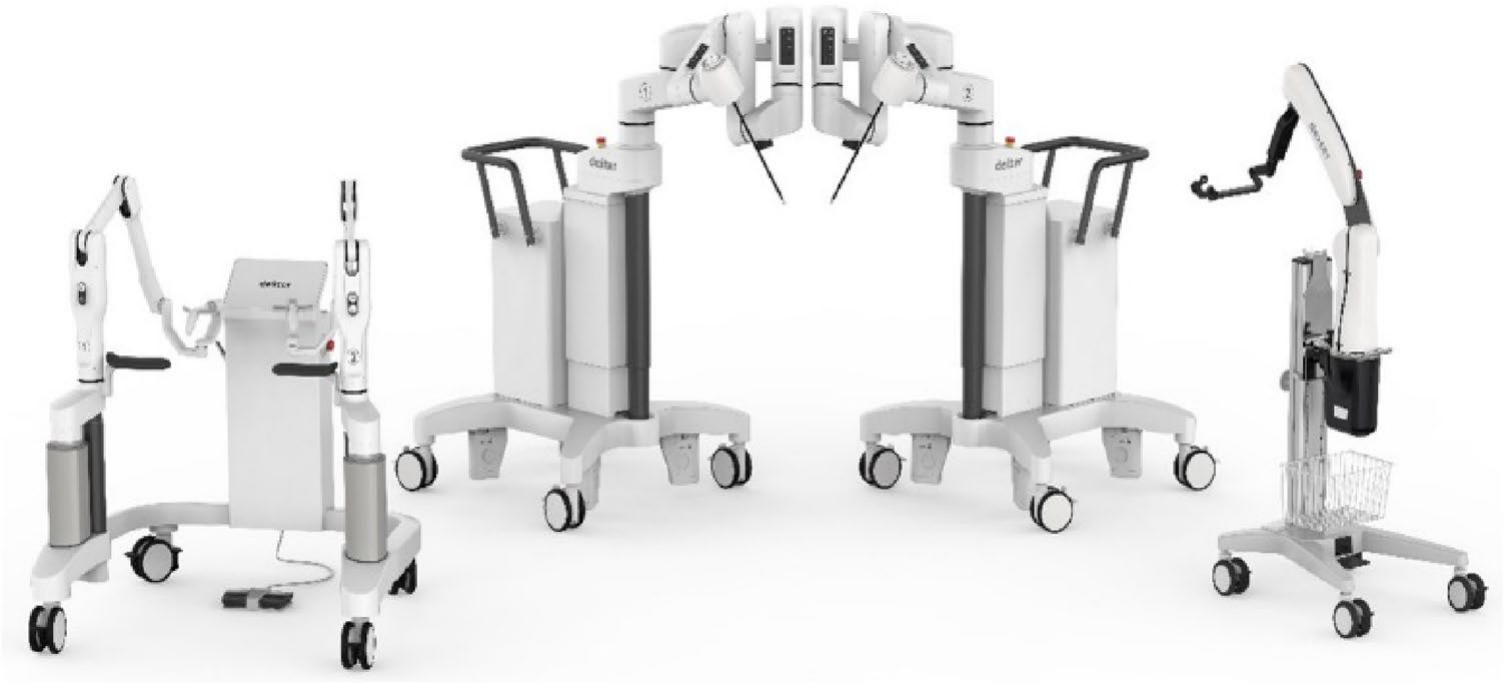
Dexter, Distalmotion. Provided by and permission from the company

**Fig. 14:**
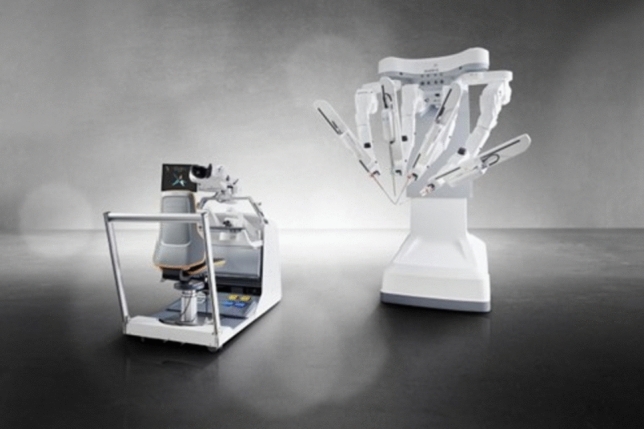
Avatera® System, avateramedical GmbH, provided by and permission from company

**Fig. 15:**
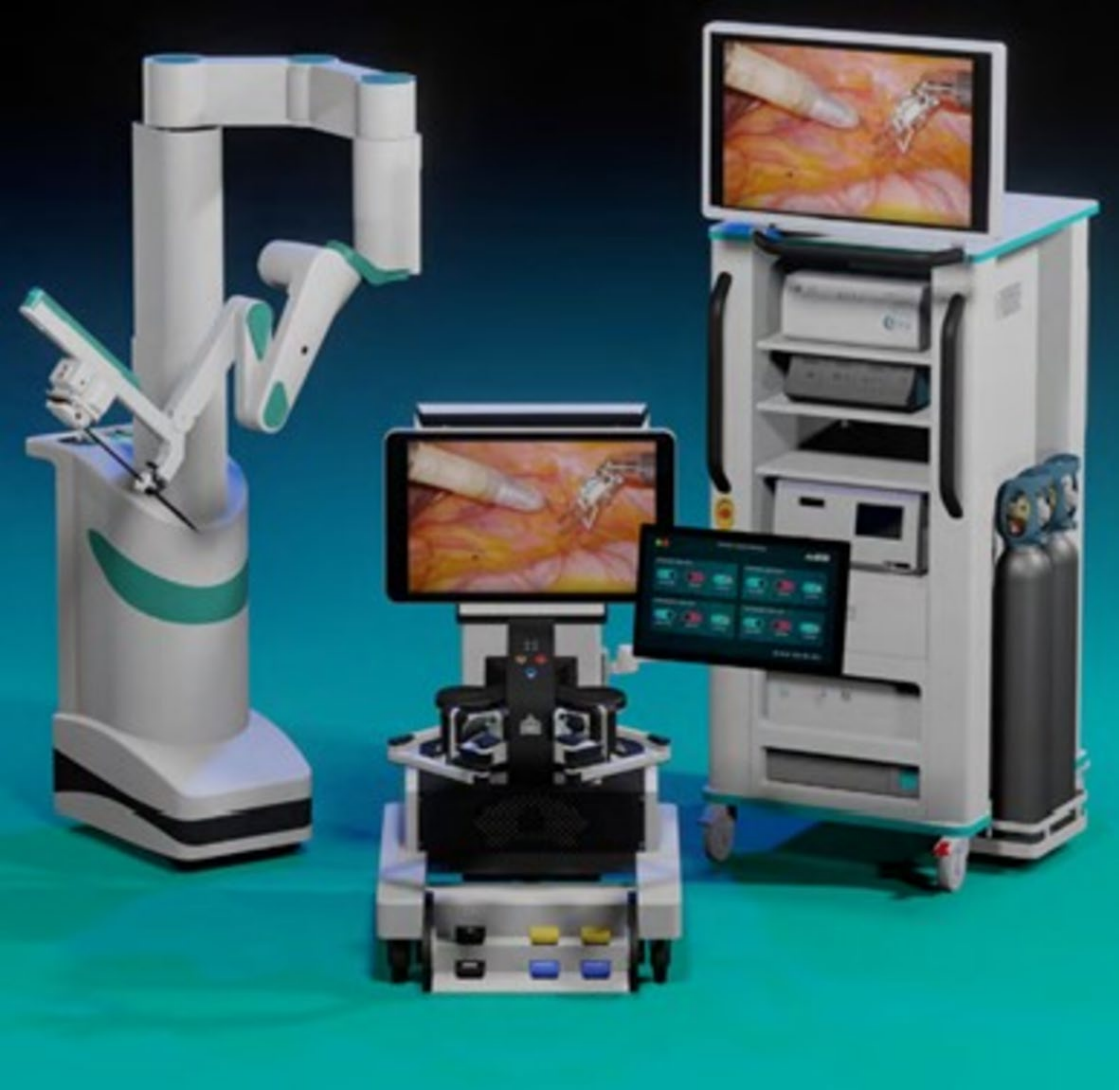
Mantra Surgical Robotic System, M/S. Sudhir, Srivastava Innovations (SSI) PVT. Ltd. Photos from product brochure with permission

**Fig. 16:**
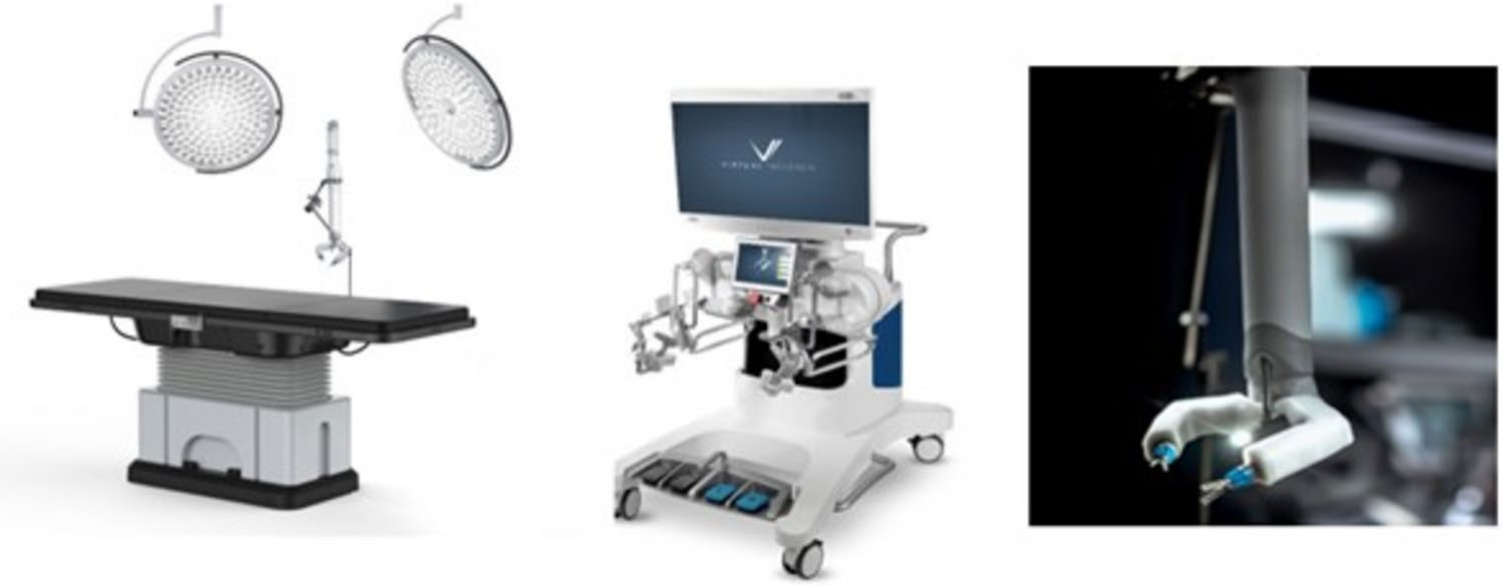
The MIRA Surgical System, Virtual Incision Corp. Images provided from company with permission and modified for the publication to fit size

**Fig. 17:**
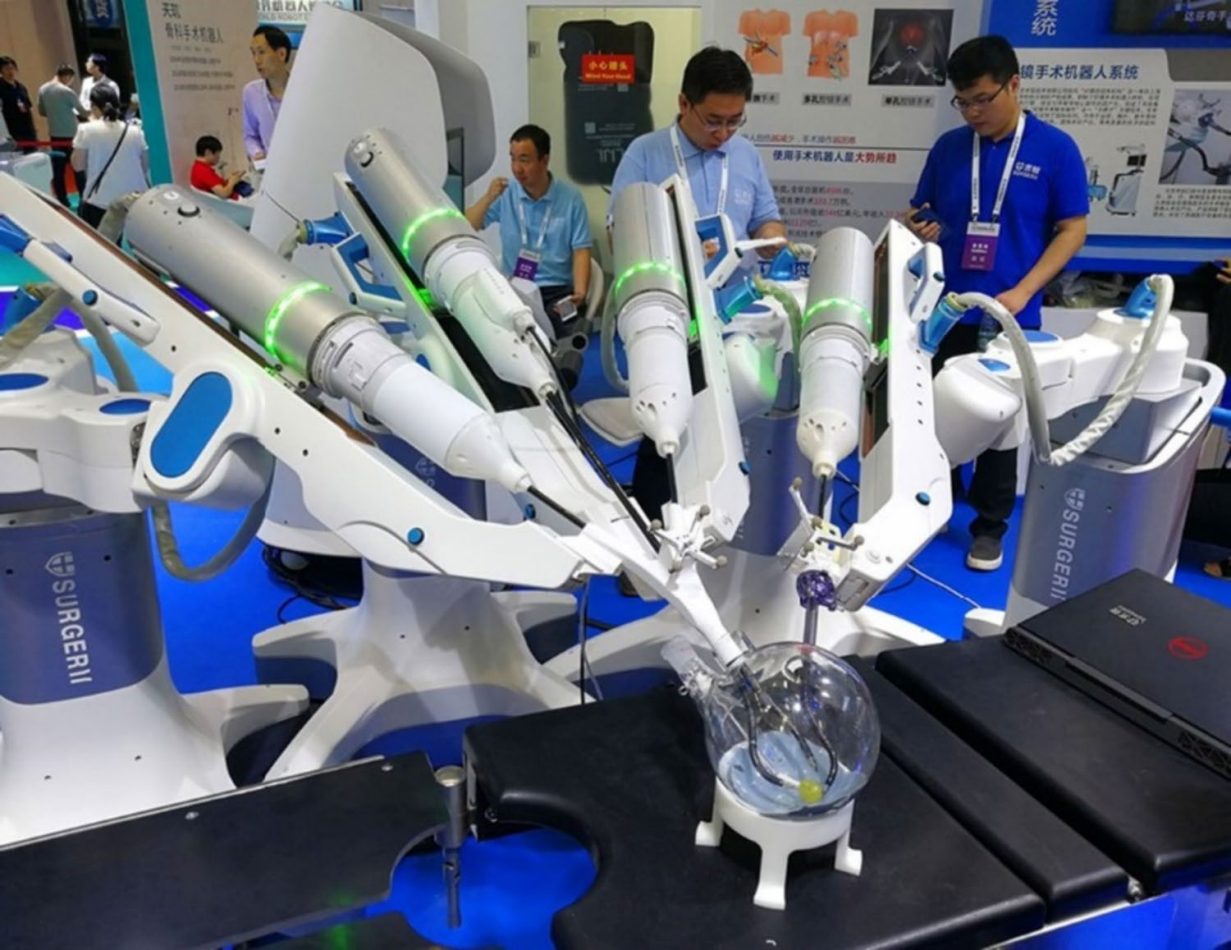
Shurui Robot, Beijing Shurui Technology Co., Ltd [106]

**Fig. 18:**
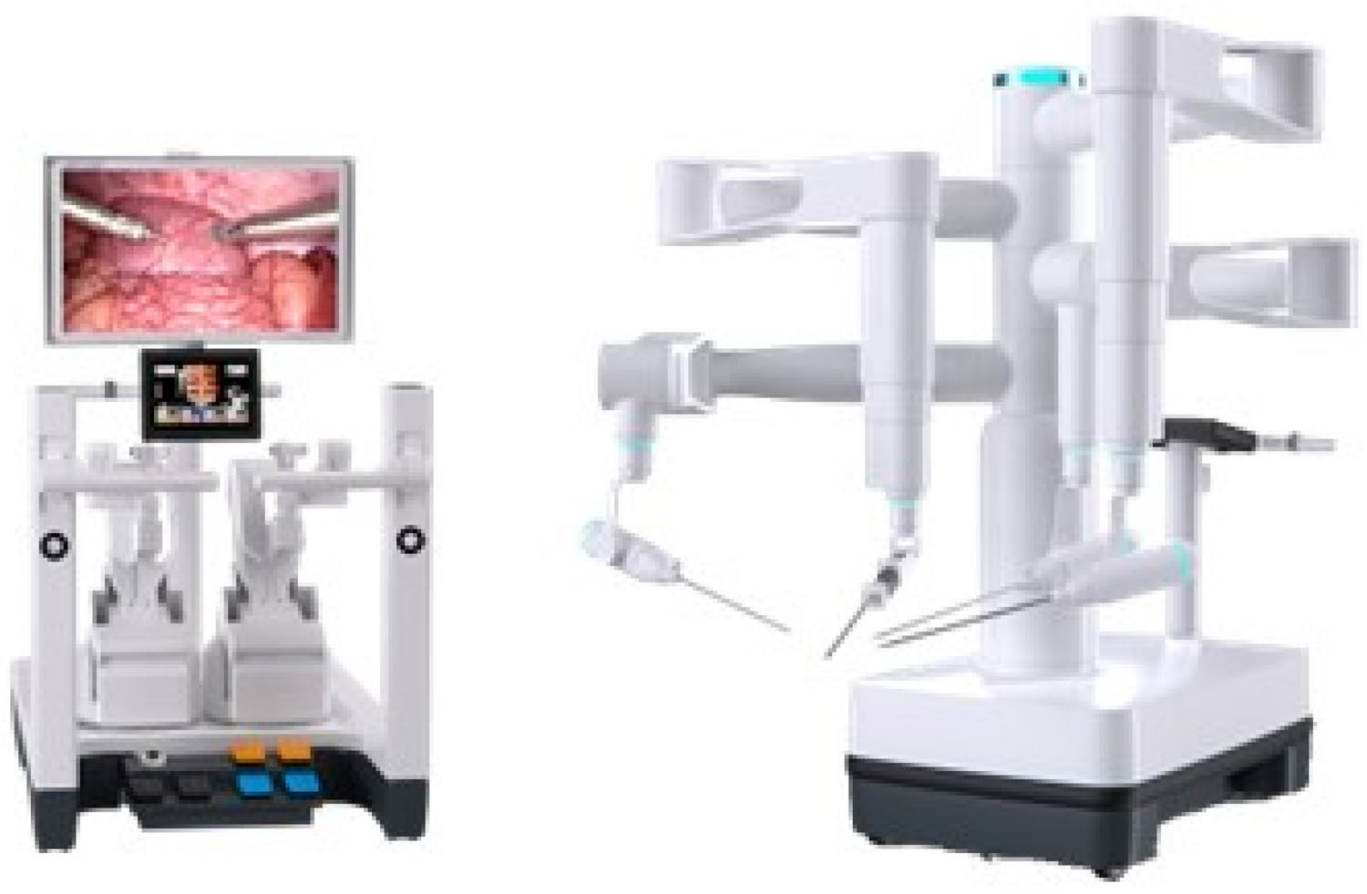
Bitrack System, RobSurgical [93]

**Fig. 19:**
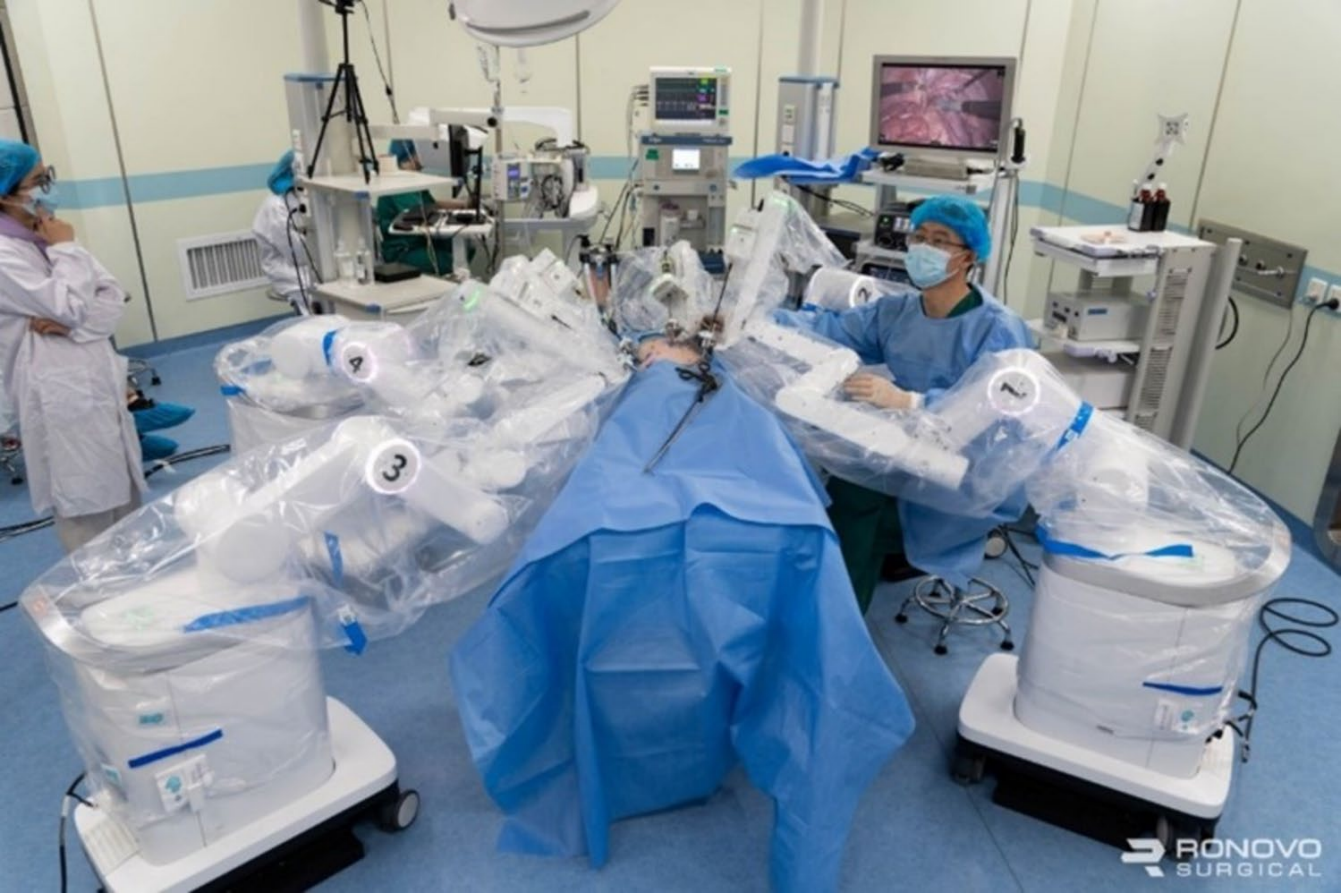
Carina, Ronovo Surgical [94]

**Fig. 20:**
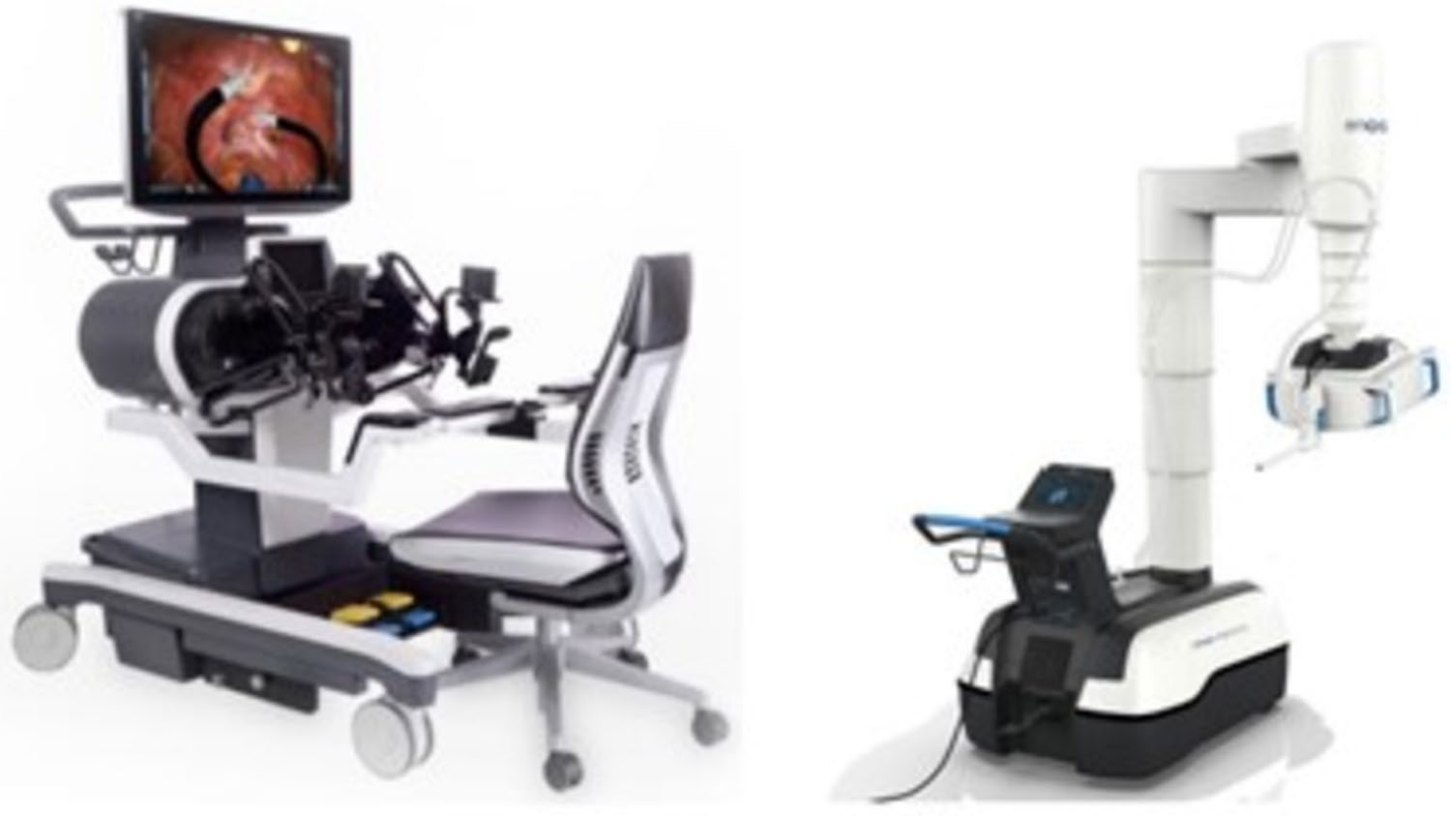
Enos™, Titan Medical Inc. Photo from website and permission from the company

**Fig. 21:**
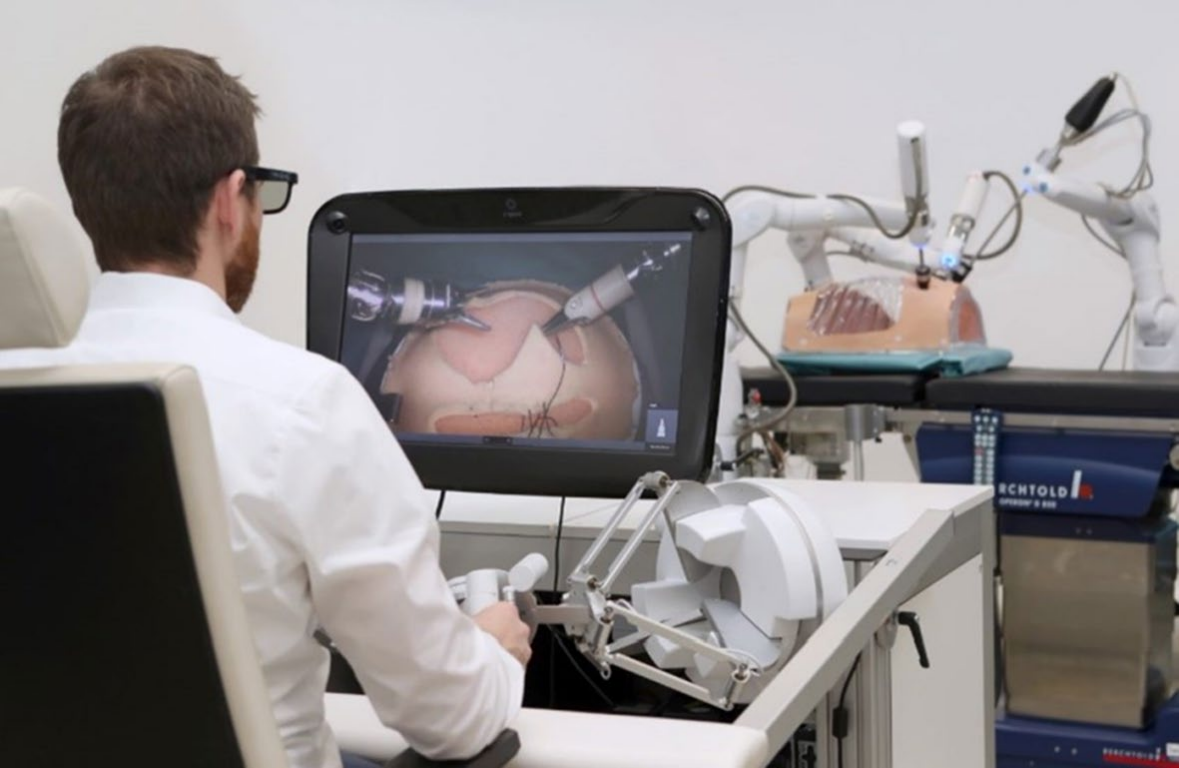
MiroSurge, DLR/Alexandra Beier (CC BY-NC-ND 3.0). Photo and permission provided by company

**Fig. 22:**
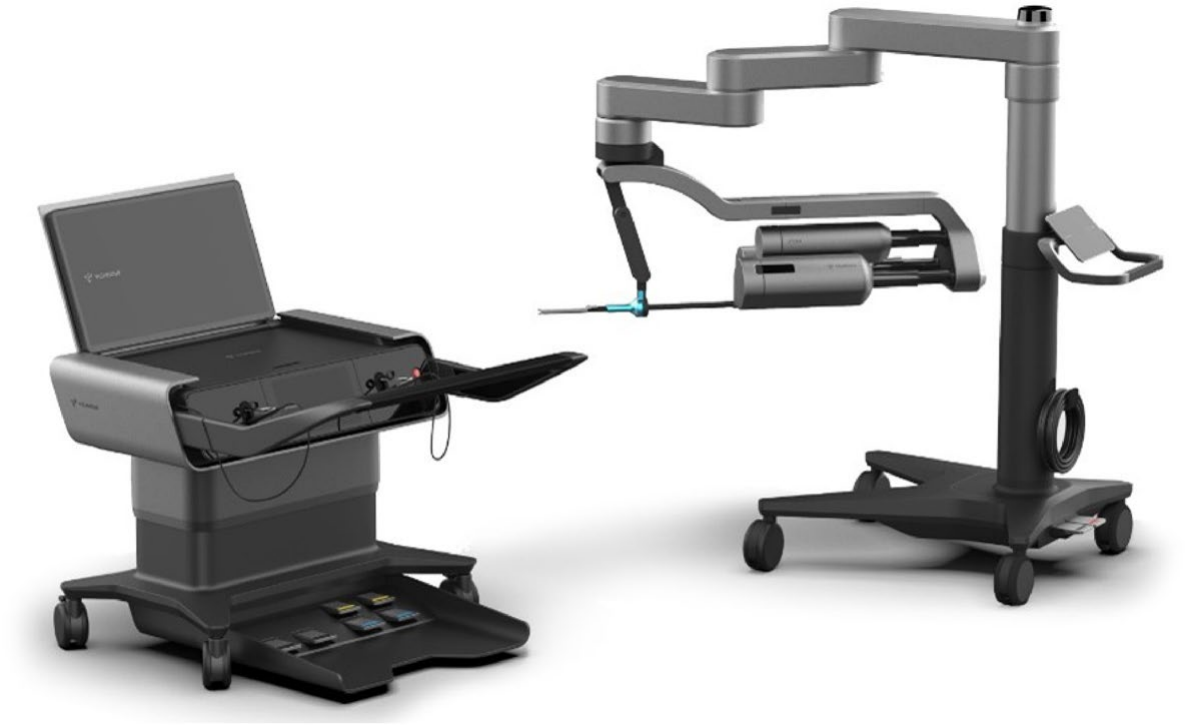
Vicarious surgical system available online via media kit

Twenty companies were approached via email for virtual interview. Twelve replied, seven met virtually and five via email. Eight companies did not reply and one there was no available email, therefore, the corresponding author of a review paper was approached, again, with no reply. Instead, for these companies, information was collected solely from the public domain.

Of these companies, China and USA have six different platforms each, Germany with two and the UK, Canada, Spain, Republic of Korea, Japan, Switzerland and India all with one platform. There was a total of 23 surgical robots for analysis. Fifteen of 21 companies represent multiport robots, four single ports, and two with both. Of the multiport systems, eight were modular and nine had a single-unit patient console.

Thirteen robotic companies have national or international regulatory approvals, with eight having none, although one robot, DLR MiroSurge from Germany, will never be used in the clinical setting.

All supporting evidence on robotic systems have been reported only in urology, general surgery and gynaecology.

Of the 17 multiport systems, one is fully evaluated at stage 4, two are stage 3, six stage 2b, two stage 2a, two stage 1, and four at the pre-IDEAL stage 0. Of the six single-port systems none have been fully evaluated with one at stage 3, three at stage 1 and two at stage 0. Pooling the 23 systems together; six have been evaluated at stage 0, and 13 at stage 1 to 2b, with four at stage 3 to 4.

Long-term data was reported for three companies. Intuitive Surgical Inc, and Suzhou Kangduo Robot Co., Ltd robotic systems both had randomised control trial data supporting evidence, whilst Asensus Surgical Ltd., have formed a multispecialty registry.

Comparison data is reported for robotic devices and platforms (Table 1). Two studies, a systematic review [21] and meta-analysis [22], found that the da Vinci single port compared to multiport, had reduced time with the catheter post prostatectomy [22], reduced length of hospital stay and opioid/analgesia administration, with equivalent oncological and continence outcomes [21, 22]. The KANGDUO Robot® Surgical System is compared to the da Vinci Si system in two studies, one on robotic assisted radical prostatectomy (RARP) demonstrating comparable short-term functional and oncological outcomes, but longer operating times in 16 patients [41]. The second, a two-centre blinded randomised control trial, showed non-inferiority in robotic assisted partial nephrectomy (RAPN) for T1a renal tumours, but with longer docking and suturing times [42]. The Revo-i system was compared to da Vinci Si in RARP, producing similar short-term functional and oncological outcomes [69]. MicroHand Surgical Robot was compared to da Vinci, reporting shorter length of stay and reduced hospital costs in 45 patients undergoing sigmoidectomy, although did not specify the da Vinci generation [63]. It also demonstrated no difference in faecal continence following total mesorectal excision when compared to da Vinci Si [64] (Table 2).

**Table 2:** Key messages

Key messages	
1. There is no uniform search strategy that can identify novel robotic platforms 2. There is a need to streamline information about various platforms and their implementation stage across different countries and languages 3. EAES could potentially be the hub to host information on current and emerging robotic devices with regular updates of their evaluation status 4. Implementation of The IDEAL Framework should be used to report evaluation of devices 5. Initial reports in the pre-clinical settings are often not published 6. High-quality studies including RCTs are required to demonstrate the true impact of the technology 7. Long-term outcome data are scarce and necessary for surveillance to ensure patient safety 8. Evaluation across specialties is necessary to demonstrate external validity 9. There is a role for societal registries to pool data on robotic platforms including long-term outcomes 10. Continued collaborative work between industry and clinicians is required	

Comparing total costs, da Vinci X and Xi is reported to be at $1.2 and $2 million respectively and an average cost per operation of $2500 [17], although clearly this will have a significant range. Other comparable systems are touted to be cheaper with KANGDUO at $1 to 1.4million, with no comparable clinical data to X and Xi systems, only Si. The Senhance® Surgical System is stated to cost between $1 to 1.2 million [17], with per procedure comparisons with da Vinci stated to be cheaper in one study, $559 versus $1393, and comparable operative times [44]. Hinotori™ Surgical Robot System and Hugo™ RAS surgical system both state that their system is cheaper than the Xi, and the avatera® system has been quoted at $1.1 million [84].

## Discussion

Our review has highlighted that full evaluation for robotic platforms has not been reported even on established robots, with the majority currently validated at stages 0 to 2b. Understandably, Intuitive is the only platform which has been fully evaluated, as it has had over twenty years to achieve long-term outcome, including randomised control trial, data. Publication of full evaluations for other systems is eagerly awaited.

The lack of evaluation reports represents a challenge for the surgical community given the rapid adoption of new systems. This situation, however, is likely to improve with time due to emerging platforms becoming commercially available.

We have provided an initial, comprehensive analysis of the platforms in this review, using The IDEAL Framework. Its intended use is for the evaluation of new, complex treatments within surgery through a logical, methodical pathway. Professor McCulloch (Chair of IDEAL) and the IDEAL team offer an explanation that competition, in this case between robotic companies, can often drive rapid adoption without full evaluation as defined by the framework. Although, safe evaluation exists with regulatory approvals before implementation into the clinical setting, it is possible that devices are introduced too quickly and not fully evaluated for certain procedures, given the increasingly competitive industry. On the other hand, it is worth noting that it would not be possible to reach stage 4 of evaluation without a platform being used in the clinical setting. Other explanations for rapid adoption pertain to the feasibility of performing multiple evaluations, across many different types of operations, within and between specialties. This would require considerable time and is unlikely to be cost-effective for robotic companies to wait for full evaluation [15]. Ultimately this would lead to the failure of bringing many platforms to market and the undesirable outcome of hindering technological progress within surgical specialties. It is also recognised that attitudes and process in healthcare differ worldwide including the adoption of new technology, therefore, evaluation of these will as well. However, broadly speaking clinicians should evaluate outcomes in the same way as IDEAL suggests i.e. through case report and series, prospective observational studies, randomised control trials against the gold standard, and long-term follow-up. Therefore, with rigorous regulatory approval and sound methodology from stage 0 to 2a, implementation of new and emerging robotic platforms is likely to be safe. Regarding long-term outcomes, multicentre, international registries could be an alternative solution to provide large data on evaluation across platforms and specialties with the European Association of Endoscopic Surgery (EAES) well positioned to provide this function for its members and beyond.

Another consideration when discussing further research within this area is whether it could distract or deviate finite resources from other fields in need. However, given that the IDEAL Framework evaluation relies on studies investigating clinical outcomes, the research required is likely to be transferable.

Several comparative studies were highlighted in Table 1, looking at clinical outcomes, however, there is greater research needed in this field. Studies are limited in scope, are often not independent from funding or involvement from the robotic company and none compare their systems to the fourth generation of Intuitive robots which are predominantly in use. Equally, these studies should not be ignored as often they demonstrate non-inferiority to the da Vinci Si i.e. safety of their use and clinical efficacy.

Considering the costs of each system it appears that the da Vinci Xi is the most expensive, but it is perhaps difficult to compare, with Intuitive Surgical Inc. producing its fourth generation. In fact, some of the systems highlighted have been created to have different capabilities and accessibility, therefore will be cheaper, but not comparable. For example, the Revo-I robot has been developed to do just this, improve accessibility, and is currently being used in Uzbekistan. It is also important to note that some of the costings quoted in the table were released by other companies or in news articles, so the reliability of this should be questioned. Lastly, details for the cost of many systems were not publicly available.

The environmental impact of robotic surgery is another important consideration. A systematic review [107] reported that robotics compared to laparoscopy had 43.5% greater greenhouse gas emissions and 24% higher waste production. Many, but not all, of the robotic systems highlighted have reusable instruments (Table 1) which will undoubtedly help to offset this. Current and emerging robotic companies should take the environmental impact of their product into account, especially for future generations of robot. This should extend beyond the procedure itself, into a more holistic approach of the perioperative pathway.

This study has a number of limitations. Firstly, although we have carried out a comprehensive search through various channels, there is a chance we may have missed emerging platforms. Evaluation is also a dynamic process and requires regular updates to provide a true account of platforms’ status.

Challenges were observed in performing a comprehensive search strategy to identify new systems, including a lack of visibility for some. Reports were occasionally not found despite being mentioned on a company’s website, making it difficult to ascertain the stage of evaluation. We had to utilise multiple resources including a literature and Google search, screening old reviews, technology articles and reaching out multiple times to company emails. These efforts are not feasible outside the research setting and it is unrealistic to expect a practicing surgeon to investigate new devices or platforms to this level of evaluation, in order to provide the user guidance.

The IDEAL Framework stage of evaluation has been awarded based on studies investigating only a limited number of operation types. There is an argument to evaluate and assign an IDEAL Framework stage for each operation type, as it would certainly differ. This topic deserves discussion and expert consensus on how to evaluate new surgical technologies and/or how the IDEAL Framework should be implemented.

It has also been argued that the framework is not optimally suited for the evaluation of future robotic systems [16]. Despite this, it is likely the best framework available which can be adapted to evaluate new technology, providing a standardised and quality-assured pathway. Importantly, the framework has been globally accepted to ensure the safe implementation of novel interventions.

## Conclusion

The majority of existing robotic platforms are currently at the preclinical to developmental and exploratory stage of evaluation. Using the IDEAL framework will ensure that emerging robotic platforms are fully evaluated with long-term data, to inform the surgical workforce and ensure patient safety.

## Data Availability

Data can be made available upon reasonable request to the corresponding author.
